# The sacrificial inactivation of the blue-light photosensor cryptochrome from *Drosophila melanogaster*†
†Electronic supplementary information (ESI) available. See DOI: 10.1039/c8cp04671a


**DOI:** 10.1039/c8cp04671a

**Published:** 2018-11-12

**Authors:** Roger Jan Kutta, Nataliya Archipowa, Nigel Shaun Scrutton

**Affiliations:** a Manchester Institute of Biotechnology (MIB) and School of Chemistry , The University of Manchester , 131 Princess Street , Manchester , M1 7DN , UK . Email: Nigel.Scrutton@manchester.ac.uk

## Abstract

*Drosophila melanogaster* cryptochrome functions as the primary blue-light receptor that mediates circadian photo entrainment. Absorption of a photon leads to reduction of the protein-bound FAD *via* consecutive electron transfer along a conserved tryptophan tetrad resembling the signalling state required for conformational changes and induction of subsequent signalling cascades. However, how the initial photochemistry and subsequent dark processes leading to downstream signalling are linked to each other at the molecular level is still poorly understood. Here, we investigated in detail the initial photochemical events in DmCRY by time-resolved and stationary absorption spectroscopy combined with quantum chemical and molecular dynamics calculations. We resolved the early events along the conserved tryptophan tetrad and the final deprotonation of the terminal tryptophanyl radical cation. These initial events lead to conformational changes, such as the known C-terminal tail release, Trp decomposition, and finally FAD release providing evidence that DmCRY does not undergo a photocycle. We propose that light is a negative regulator of DmCRY stability even under *in vitro* conditions where the proteasomal machinery is missing, that is in line with its biological function, *i.e.* entrainment of the circadian clock.

## Abstract 2

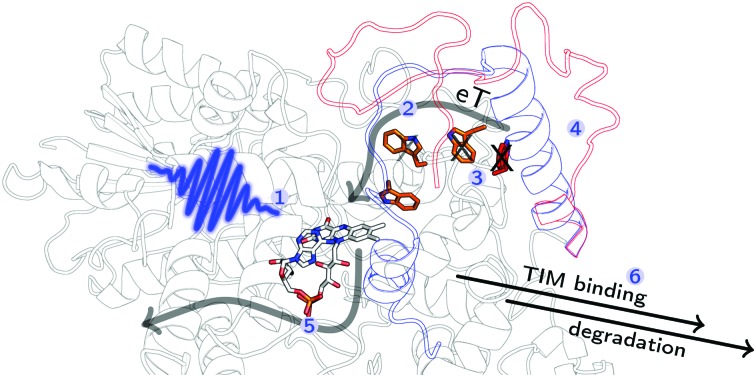
Photoactivation of *Drosophila melanogaster* cryptochrome results in tryptophan decomposition, conformational changes, and final FAD release.

## Introduction

Photosensory proteins play a crucial role in a wide range of biological systems. So far, eight groups of biological photosensors have been identified with functions ranging from the initiation of simple physiological functions in bacteria over the control of morphogenesis and development in plants to the enormously complex process of vision in vertebrate animals.^1^–^3^ The actual photon sensor is the chromophore that absorbs light and transmits that energy to the protein backbone resulting in the activation of the photosensor, *e.g.* by conformational changes, and the initiation of a signalling cascade.

**Fig. 1:**
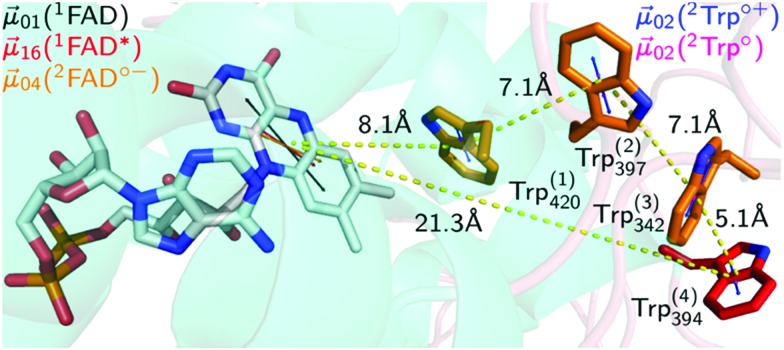
Electron transfer cascade in DmCRY. Cartoon representation of FAD (grey carbons) bound to DmCRY and the adjacent conserved tryptophan tetrad (orange/red carbons) based on DmCRY's crystal structure (pdb code: 4GU5).^14^ All distances (yellow dashed lines) are measured from the centre of mass of each moiety. Additionally, the transition dipole moments of the most prominent absorption transitions are shown as indicated in the legend.

A wide variety of signalling functions, *e.g.* regulation of the circadian clock throughout nature,^4^ growth and development in plants,^5^ and putative role as magnetoreceptor in animals,^6^ can be found in the widespread protein family of cryptochromes (CRY). In terms of circadian context animal CRY are subdivided into two classes of proteins: the light-responsive type I (invertebrates) and the light-independent type II (mainly vertebrates).^7^ With the exception of animal type II CRY that can mainly be found in vertebrates,^8^ CRYs are defined as flavoprotein blue-light photosensors containing the cofactor flavin adenine dinucleotide (FAD) in the fully oxidised redox form.^7^ CRYs are closely structurally related to photolyases, the ancient light-dependent DNA repair enzymes, with an additional C-terminal tail (CTT) which is responsible for mediating phototransduction.^7^ In the fruit fly, *Drosophila melanogaster* (Dm), light was shown to induce conformational changes in the CRY CTT,^9^,^10^ enabling interaction with the core clock protein TIM.^11^ Binding of DmCRY to TIM leads to ubiquitination and proteasomal degradation of both proteins^12^,^13^ that is required for clock entrainment. However, how the initial photochemistry and subsequent light-independent processes leading to downstream signal transduction are linked to each other in CRY at the molecular level is still poorly understood. The initial photochemical events that lead to DmCRY conformational changes were extensively studied *in vitro* and are proposed to be as follows (Fig. 1).

Upon photon absorption of protein-bound FAD fast electron transfer (eT) takes place from a nearby tryptophan (Trp) to the excited singlet state of FAD^15^ forming an ionic radical pair (RP) [FAD˙^–^,TrpH˙^+^] within *ca.* 1 ps in animal type I CRY^16^,^17^ and between 0.4 and 0.8 ps in plant CRY and photolyases.^16^,^18^–^21^ This RP can either recombine back to the corresponding ground state species within *ca.* 30 ps,^16^ or can undergo charge separation *via* further eT along a conserved so-called Trp-triad/tetrad^22^–^24^ until the terminal surface exposed tryptophanyl radical cation (TrpH_394_˙^+^) is formed. This ultrafast eT occurs on a ps timescale as has been shown for some photolyase/CRY family members, *e.g.* within 30 ps in *E. coli* photolyase, on a 100 ps time scale in an algal CRY, and 60–220 ps in animal type I CRY.^16^,^18^–^21^,^25^ The RP [FAD˙^–^···TrpH_394_˙^+^] is further stabilised by deprotonation of TrpH_394_˙^+^ occurring with a lifetime of 2.6 μs.^26^ No further reduction of FAD˙^–^ in the case of animal type I CRY was observed. It is supposed that the RP [FAD˙^–^···Trp_394_˙] undergoes recombination with a lifetime of 6.8 ms.^26^ However, significant yields of FAD˙^–^ are observed on a stationary level which disagrees with a RP lifetime of 6.8 ms. The FAD˙^–^ re-oxidation is strongly oxygen dependent, occurring on a second to minute timescale.^27^–^30^

Here, we investigated the initial photochemical events in DmCRY by time-resolved and stationary absorption spectroscopy in the visible range combined with quantum chemical and molecular dynamic calculations in order to provide a molecular link between the initial photochemistry and subsequent dark processes leading to downstream signal transduction.

## Experimental

### Expression and purification of CRY

The DmCRY (UniProtKB-O77059) samples were expressed and purified as previously described.^8^ The samples were dissolved in 50 mM HEPES buffer (pH = 8.0) containing 150 mM NaCl and 10% glycerol for time-resolved UV/Vis absorption measurements or 20 mM Na_2_HPO_4_, 50 mM NaCl and 10% glycerol buffer (pH = 8.0) for stationary UV/Vis absorption or circular dichroism measurements. All measurements were performed at room temperature (*ca.* 20 °C).

### Stationary UV-Visible absorption spectroscopy

Stationary UV/Vis absorption spectra were recorded using an Agilent Cary 50 UV/Vis Spectrophotometer. A sample of DmCRY was illuminated using a pulsed, high power light emitting diode (LED; M455L3, Thorlabs) with *λ*_max_ = 455 nm. The 1 s rectangular excitation pulse (20 mJ) was collimated using an anti-reflection coated aspheric lens (Thorlabs) and delivered along the 2 mm pathlength of the cuvette orthogonal to the detection beam. After excitation an absorption spectrum was recorded directly afterwards. The sample volume was 120 μL with an optical density over 1 cm of 0.25 at the excitation wavelength. Thus, the entire sample volume was homogeneously illuminated, avoiding possible effects of diffusion during the recording of the spectra.

In the case of repeated illumination experiment, the sample was incubated in darkness for *ca.* 15 min between the light pulses to give enough time for flavin re-oxidation until no changes were observed in the UV/Vis spectra. After full re-oxidation and prior the next blue-light pulse, the sample was subjected to stationary circular dichroism absorption spectroscopy. The total illumination time was 30 s with 10 × 1 s, 2 × 5 s and 1 × 10 s blue-light pulses. Anaerobic samples were prepared in a glove box (Belle Technology, UK Ltd) by incubation overnight at ambient temperature in darkness. For comparison an aliquot of the sample was also incubated overnight outside the glove box under otherwise identical conditions.

### Time-resolved UV-Visible absorption spectroscopy from ms to s

Analogous to a laser flash experiment, a commercial UV-Visible spectrometer (Varian, Cary 50) was used to cover the dynamics in the millisecond to seconds dynamics of DmCRY. The sample was excited orthogonally by a pulsed high-power LED at 455 nm (M455L3, Thorlabs) with a flash of 10 ms duration and 20 mJ pulse energy, which was collimated by an anti-reflection coated aspheric lens (Thorlabs) to yield a pump fluence of approximately 10 mJ cm^–2^. The LED flash was synchronized with the spectrometer recording. A small sample volume of 120 μL with an OD of ∼0.1 over 10 mm at the excitation wavelength was used, allowing excitation of the entire sample volume to avert diffusion effects on the long timescales measured. The absorbance changes were recorded at single wavelengths ranging from 400 to 700 nm in 10 nm steps with a time resolution of 12.5 ms in a time window of 10 s.

### Stationary circular dichroism (CD) absorption spectroscopy

Stationary circular dichroism absorption spectra were recorded using an Applied Photophysics Chirascan CD Spectrophotometer at 20 °C. In the UV range from 180 to 350 nm a pathlength of 100 μm and in the visible range from 350 to 600 nm a pathlength of 10 mm was used. Illumination conditions are described above. The buffer background was subtracted from each spectrum. Fig. 4c and d show an average of 5 scans, respectively.

### Sub-ps pump/supercontinuum-probe spectroscopy

A Ti–sapphire amplifier system (Spectra Physics Solstice Ace) was used to generate 800 nm with 6 mJ pulses at 1 kHz. One third of the 800 nm pulses were used to pump a collinear Optical Parametric Amplifier (OPA, TOPAS-PRIME, Light Conversion) with an associated NirUVis unit tuned to pump pulses centred at *ca.* 450 nm (*ca.* 10 nm full width at half-maximum (FWHM), *ca.* 100 fs, *ca.* 150 nJ). The supercontinuum white light probe pulses were generated by focusing a small fraction of the 800 nm pulses into a moving CaF_2_ disc of 3 mm thickness giving a probe spectrum ranging from 340 to 820 nm. For the sub-ps time range a Helios spectrometer (Ultrafast Systems) was used equipped with a delay line up to 3 ns. Two complementary fiber coupled high-speed spectrometers equipped with metal–oxide–semiconductor (CMOS) detectors for signal and reference recording were used. The pump and probe pulses were focused colinearly into the sample to spot sizes of *ca.* 200 μm and 100 μm FWHM, respectively. The relative polarizations between the pump and probe were set by a half-wave plate in the pump-beam path to magic angle (54.71°) for observations of pure population changes or to either parallel (0°) or orthogonal (90°) for observation of the anisotropy. The averaged pre-t_0_ laser scatter signal was subtracted from the data and the *ca.* 1.5 ps chirp of the white light was corrected for prior to data analysis using the coherent artefact as an indicator for time zero at each wavelength. Throughout the probe range, the spectral resolution was better than 4 nm and the temporal resolution was better than 100 fs. 10 individual scans with averaging 100 spectra per time point were typically recorded. The time axis – within total 500 points – was linear between –1 and 1 ps and logarithmic from 1 ps to the maximum time delay so that the same number of delay points from –1 to 1 ps as between 1 and 10 ps, 10 and 100 ps, *etc.* is produced ensuring that the dynamics on every timescale will have equal weighting in the fitting analysis. Samples were stirred inside the cell with a path length of 2 mm for pump and probe beams (dimensions: 10 mm × 2 mm × 30 mm, Starna). 3 independent measurements with 10 scans along the delay stage with 100 ms integration per spectrum and per time-step were performed and analysed independently. In all cases all scans resulted in reproducible data sets. Additionally, the integrity of the sample was checked by recording stationary absorption spectra before and after each measurement. Tryptophan photo-product formation was kept to a minimum under the used experimental conditions, *i.e.* increase of absorption at 315 nm by less than 34%. The shown data correspond to one representative measurement. No smoothing or filtering procedures were applied to the data.

### ns to ms transient absorption spectroscopy by using a streak camera set-up

The streak camera set-up was used as described previously.^31^,^32^ In brief, the third harmonic of a Nd:YAG laser (10 Hz, Surelite II, Continuum) pumping an Optical Parametric Oscillator (OPO, Continuum) tuned to 450 nm (10 mJ, *ca.* 10 ns) was used for sample excitation. As a probe light a pulsed 150 W Xe-flash lamp (MSP-05, Müller Elektronik-Optik) was used which was focused three times *via* toric mirror optics: (i) before probe shutter, (ii) into sample, (iii) into spectrograph. The entire white light probe pulse was analysed by a combination of a spectrograph (200is, Bruker) and a streak camera (C7700, Hamamatsu Photonics). The use of mechanical shutters enabled the recording of a sequence of three individual data sets: (i) an image (*D*_FL_) with both flash lamp and laser, (ii) an image (*D*_0_) without any incoming light, and (iii) an image (*D*_F_) only with the flash lamp. 100 of such sequences were recorded and corresponding data sets were averaged. Then, the TA was calculated as:1
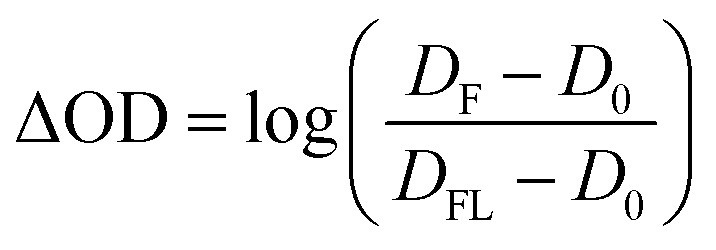
A 10 mL sample was stepwise cycled by a peristaltic pump (Pump P-1, Pharmacia) through a flow cell with a pathlength of 2 mm for pump and 10 mm for probe beams (dimensions: 2 mm × 10 mm × 30 mm, Starna) ensuring a total replacement of the sample prior to each individual measurement.

### Transient absorption data analysis and modelling

SVD-based rank analysis and global fitting were performed using an in-house written program described previously.^31^,^32^ In very short, the linear least squares problem in eqn (2)

2 *χ*^2^ = ||Δ**A** – **FB**||^2^ = Min

is solved, where Δ**A** is the time-resolved absorption data matrix, **F** is the matrix containing the analytical functions accounting for the temporal changes in the data, *i.e.* exponential decays (convoluted with the instrument response, typically a Gaussian function), and **B** is the matrix with the to be determined spectra. Further optimization of *χ*^2^ is achieved by optimizing the rate constants in **F** by a nonlinear least squares algorithm. As a result of such fits so-called decay associated difference spectra (DADS in matrix **B**) and their associated optimized rate constants are obtained. These are the unique result of this global fit and this treatment does not require any model for the kinetics involved in the transient processes. The number of exponentials to use in the global fit is determined by SVD-based rank analysis, which is described elsewhere.^33^ The model that relates the actual species kinetics to the elementary function is applied afterwards resulting in species associated spectra (SAS). The shape of the SAS in terms of identity with well-known spectra or following physical laws decides about the appropriateness of the model. This step does not change the *χ*^2^ value found in the global fit and, therefore, this procedure has the advantage that all interpretation is performed with the same quality of fit.

As an alternative analysis, known species spectra, taken either from literature or recorded in this work, were taken in order to decompose the recorded time-resolved data matrix using the transpose of the data matrix in eqn (2) and using the basis spectra instead of analytical functions. The resulting concentration time-profiles inform about the appropriateness of the basis spectra and the physical reasonability, *i.e.* total sum of species being constant to 1.

### Quantum chemical calculations

Quantum-chemical calculations on all molecular moieties that potentially contribute to the time-resolved absorption signals in the DmCRY photochemistry, *i.e.*^1^FAD, ^1^FAD*, ^2^FAD˙^–^, ^2^Trp˙^+^, ^2^Trp˙, ^2^Tyr˙^+^, and ^2^Tyr˙, were performed using the Firefly QC package,^34^ which is partially based on the GAMESS (US)^35^ source code. All ground state structures were optimized on the level of restricted open shell density functional theory (ROHF-DFT) using the B3LYP functional and the split-valence 6-31G(p,d)++ basis set. The structure of the first excited singlet state of flavin was optimized on the level of restricted closed shell time dependent density functional theory (RHF-TD-DFT) using the B3LYP functional and the split-valence 6-31G(p,d)++ basis set. Complete active space self-consistency field (CASSCF) theory was used in order to calculate the static correlation energy. The most intense electronic transitions of oxidised flavin, *i.e.* S_X_ ← S_0_, first excited singlet state flavin, *i.e.* S_X_ ← S_1_, and singly reduced flavin, *i.e.* D_X_ ← D_0_ are all of π–π* type. All n–π* transitions are of minor importance and were not further considered. Thus, 14 contributing π electrons were included into the CAS, distributed over 14 molecular orbitals (MO), and energy averaging over 10 states with equal weights, *i.e.* CASSCF(14,10)10, was performed. In case of the tryptophan radicals, *i.e.* cationic and neutral, the important electronic transitions are also of π–π* type. Here, 11 π electrons were distributed over 12 MOs in the CAS, and energy averaging over 6 states with equal weights, *i.e.* CASSCF(11,12)6, was performed. In case of the tyrosine radicals, *i.e.* cationic and neutral, the important electronic transitions are also of π–π* type. Here, 7 π electrons were distributed over 8 MOs in the CAS, and energy averaging over 6 states with equal weights, *i.e.* CASSCF(7,8)6, was performed. In order to calculate the dynamic correlation energy extended multi-configuration quasi-degenerate perturbation theory (XMCQDPT) was used on top of the CASSCF optimized MOs.^36^ In all cases an intruder state avoidance (ISA) denominator shift of 0.02 was used. To note, since only π–π* type transitions were considered throughout, the numbering of the electronic states that is presented in this work may not correspond to the actual numbering found in each particular chemical species. All transition dipole moments obtained in this work agree very well with previously reported values (see Fig. S4 and S7, ESI†).^20^,^37^

### Molecular dynamics simulations

Molecular dynamics simulations using the Gromacs package^38^ were performed on wild type DmCRY (pdb code: ; 4GU5)^14^ in its ‘dark’ ground state and in its four ‘light’ radical pair states, *i.e.* [FAD˙^–^,Trp_420_^(1)^˙^+^], [FAD˙,Trp_397_^(2)^˙^+^], [FAD˙^–^,Trp_342_^(3)^˙^+^], and [FAD˙^–^,Trp_394_^(4)^˙^+^] (Fig. S1, S11, S12 and Table S1, ESI†). For completeness we also performed molecular simulations on [FAD˙^–^,Tyr_158_^(1)^˙^+^], [FAD˙^–^,Tyr_317_^(2)^˙^+^], and [FAD˙^–^,Tyr_319_^(3)^˙^+^] due to postulations of alternative eT pathways (Fig. S13 and Table S2, ESI†).^39^,^40^ The systems were parameterized based on the gromos96 ; 43a1 force field, where, as a first order approximation, the charges of the radical ions were altered in accordance to the Mulliken charges obtained from an unrestricted open shell 2nd order Møller–Plesset perturbation (UHF-MP2) calculation with aqueous solvation effects accounted by the polarizable continuum model (PCM) using the Firefly QC package,^34^ which is partially based on the GAMESS (US)^35^ source code. A solvation box of minimum 1 nm around the protein and periodic boundary conditions were used. After energy minimization, the system was initially thermalized to 300 K for 100 ps using *NVT* dynamics, and the pressure was then equilibrated for 100 ps using *NPT* dynamics. The protein and cofactor, either FAD or ^2^FAD˙^–^, were constrained during these steps. All constraints and pressure couplings were then switched off and the system relaxed using *NPT* at 250, 280, 290, and 300 K for 1 ns each. Finally, 100 ns of *NPT* dynamics were run at 300 K.

## Results and discussion

### Early events of DmCRY reaction mechanism

**Fig. 2:**
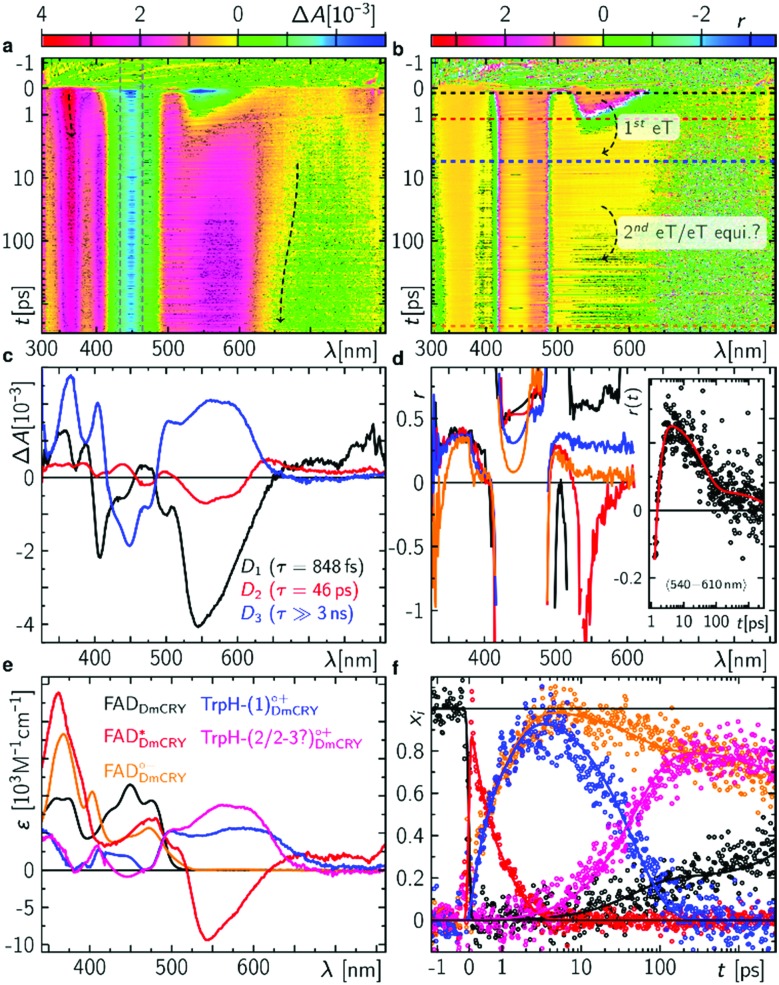
Initial events after photo excitation of FAD inside DmCRY. (a) Raw data of the time-resolved absorption spectra recorded under magic angle conditions in false colour representation (see also Fig. S3 for selected time traces, ESI†). The grey dashed rectangle indicates the data for which laser scattering was corrected. Thus, these data have a higher uncertainty than the rest of the data. (b) Corresponding time-resolved anisotropy data. (c) Decay associated difference spectra (*D*_i_, black, red, and blue) from global fit. (d) Selected anisotropy spectra as indicated in (b) by horizontal dashed lines. Inset: Anisotropy as a function of time averaged between 540 and 610 nm (black) and the corresponding tri-exponential global fit (red) using the rate constants obtained from the global fit in (c). (e) Species associated spectra used for decomposition of raw data shown in (a) (see text). (f) Decomposed mole-fraction *vs.* time profiles plus analytical solution of model shown in Fig. 6. Colour coding as in (e).

**Fig. 3:**
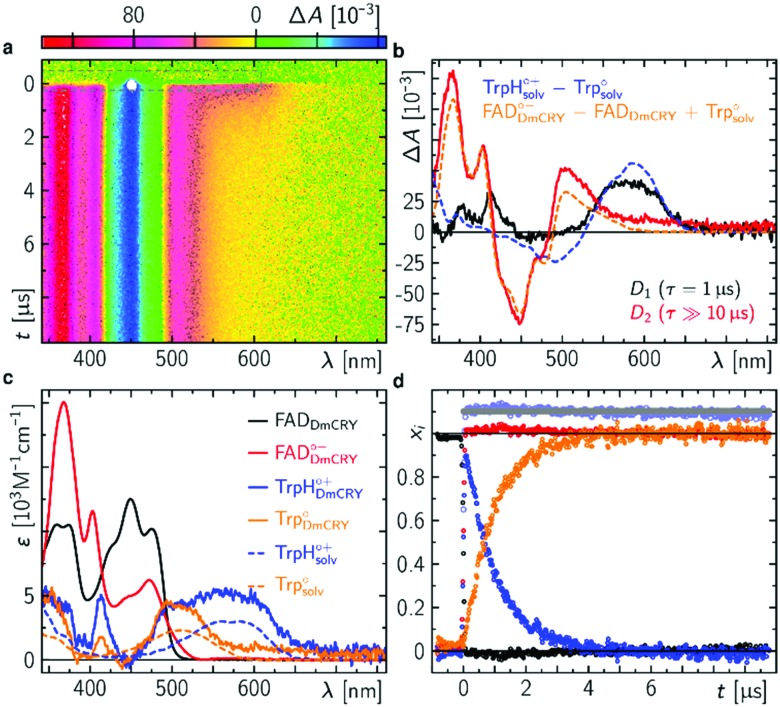
Intermediate events after photo excitation of FAD inside DmCRY. (a) Raw data of the time-resolved absorption spectra in false colour representation. The grey dashed rectangle indicates a spectral region, where the laser scatter and the small amount of flavin emission was replaced by the fit during the global fitting procedure as described elsewhere.^32^ (b) Decay associated difference spectra (*D*_i_, black and red) from global fit and linear combination spectra of known species spectra as indicated. (c) Species associated spectra (solid line) used for decomposition of raw data shown in (a) (see text). The dashed lines are reference spectra^44^ as indicated. (d) Decomposed mole-fraction *vs.* time profiles. Colour coding as in (c). Grey and pale blue circle show the sums (offset by 0.1 for visibility) of flavin and Trp contributions, respectively.

We started with an investigation of the primary events during DmCRY flavin photoreduction on the sub-ps to ns timescale under magic angle conditions (Fig. 2a) and determined the corresponding anisotropies (Fig. 2b). Initially, after excitation at 450 nm a broad transient absorption (TA) signal is observed with positive excited state absorption (ESA) peaking at 360, 500, 700, and 775 nm, negative ground state bleach (GSB), and stimulated emission (SE) features peaking at 450 nm and 545 nm, respectively (Fig. 2a). Together with the corresponding anisotropies (dashed black line in Fig. 2b) these signals resemble the well-known spectral features of the first excited singlet state of flavin, ^1^FAD*, as shown by our experimental and theoretical data (Fig. S1, S2, S4, S5, and Table S1, ESI†) and are additionally in good agreement with previous reports.^41^,^42^ Within the next 4 ps SE and ESA features at 700 and 775 nm decay accompanied by a redshift of *ca.* 5 nm of the 360 nm band (dashed arrow in Fig. 2a). Furthermore, new TA signals arise simultaneously peaking at *ca.* 405 nm and between 490 and 700 nm indicating the formation of a new electronic species. In the first 1 ns the broad TA signal between 490 and 700 nm narrows by *ca.* 50 nm from the red spectral region (dashed arrow in Fig. 2a) accompanied by a small decrease in intensity of the TA signals <410 nm. Correspondingly, on the entire experimental time window of 3 ns the GSB measured at 475 nm recovers slightly (Fig. S3, ESI†). According to our calculations and previous experimental work^18^,^43^ the spectrum of FAD˙^–^ is only slightly redshifted compared to the spectrum of ^1^FAD* in the range <500 nm (Fig. S2, S5, and S6, ESI†). Thus, the observed kinetics within the first 4 ps and corresponding anisotropies (Fig. 2b, d, Fig. S1 and Table S1, ESI†) show the formation of the FAD˙^–^ without deactivation into the ground state. Since FAD˙^–^ does not absorb strongly >500 nm the observed TA must correspond to the spectrum of the counter radical. Based on our calculations and previous experimental work^44^ the newly formed broad TA signal between 490 and 700 nm resembles the spectrum of a tryptophanyl radical cation (Fig. S7, ESI†). Taking experimental and theoretical anisotropies evaluated from more realistic relaxed structures due to protein dynamics into account (Fig. 2b, d, Fig. S1b and Table S1, ESI†) this allows the assignment of TrpH_420_^(1)^˙^+^ contributing to the TA signal arising from the initially formed RP [FAD˙^–^,TrpH_420_^(1)^˙^+^] (*r*_experiment_ ∼ 0.25 and *r*_theory_(TrpH_420_^(1)^˙^+^) ∼ 0.21 ± 0.05). The narrowing of the tryptophanyl radical cation spectrum indicates a change in environment due to further eT reactions. Considering the observed and calculated anisotropies (Fig. 2b, d, Fig. S1, S11, S12, and Table S1, ESI†), one would expect a slightly negative anisotropy (*r*_theory_(TrpH_394_^(4)^˙^+^) ∼ –0.05 ± 0.05) for the final ionic RP, [FAD˙^–^,TrpH_394_^(4)^˙^+^]. However, we observe a slightly positive anisotropy (*r*_experiment_ ∼ 0.05) accompanied by the narrowing of the tryptophanyl radical cation spectrum and a begin of another decay longer than the detection window towards an anisotropy of zero. Thus, within our detection window we can exclude the formation of the pure final ionic RP, [FAD˙^–^,TrpH_394_^(4)^˙^+^]. Therefore, the observed kinetics and accompanied anisotropies can be assigned to either the pure 2nd eT from TrpH_397_^(2)^ to TrpH_420_^(1)^˙^+^ (*r*_theory_(TrpH_397_^(2)^˙^+^) ∼ 0.16 ± 0.08) or the formation of an equilibrium state between the first three TrpH˙^+^ or a subgroup of those. Since we observe a decay of the signal of TrpH_420_^(1)^˙^+^ a hypothetical equilibrium is shifted towards the subsequent RPs, which would interconvert faster than they are formed. Thus, an equilibrium between the second and third TrpH˙^+^ and under the assumption that both radicals are equally populated the averaged theoretical anisotropy gives 0.02 (*r*_theory_(TrpH_397_^(2)^˙^+^) ∼ 0.16 ± 0.08, and *r*_theory_(TrpH_342_^(3)^˙^+^) ∼ –0.12 ± 0.04). Interestingly, for other photolyase cryptochrome family members eT reactions are proposed to be completed within 1 ns.^18^–^21^,^25^ In a next step, we quantified the kinetics of the data by a three exponential global fit resulting in the so-called decay associated difference spectra (DADS, Fig. 2c) with corresponding rate constants (848 fs)^–1^, (46 ps)^–1^, and (≫3 ns)^–1^. In general, the sum of all DADS resembles the spectrum of the initially excited species minus ground state. Thus, the spectrum of the first excited state of bound FAD can be obtained by addition of the ground state spectrum of bound FAD (Fig. S14a, ESI†) to the sum of all DADS. This defines the amount of ground state contributing to the data allowing for estimating the quantum yields for individual radical pair formations. At this stage of the analysis, models considering equilibria between the two protonation states of Trp radicals can be excluded, since in such cases the extinction coefficients of those radicals would exceed that of FAD. Furthermore, based on DADS modelling using known species spectra the involvement of the flavin neutral radical can be excluded (Fig. S15, ESI†). Then, DADS-3 with rate constant (≫3 ns)^–1^ resembles the linear combination of all none decaying species spectra in the recorded time window minus the ground state spectrum. Here, it consists of either the pure spectra of the radical pair [FAD˙^–^,TrpH_397_^(2)^˙^+^] minus ground state or the spectra of the equilibrated radical pairs [FAD˙^–^,TrpH^(2–3)^˙^+^] minus ground state. Accordingly, the subtraction of the difference spectrum between FAD˙^–^ and FAD from DADS-3 such that all flavin spectral features disappear (magenta line in Fig. 2c and Fig. S14, ESI†) results in either the pure TrpH_397_^(2)^˙^+^ spectrum or the equilibrated TrpH^(2–3)^˙^+^ spectrum. In both cases a good agreement in terms of shape with the known solution spectrum is given (blue dashed line in Fig. 3c). 80% of the difference spectrum are needed indicating 20% of radical pair recombination (RPR) in line with the small amplitude changes at <410 nm and at the GSB observed in the raw data (Fig. 2a and Fig. S3, ESI†). Since both scenarios follow the same analysis, so that addition of the resulting TrpH^(2/2–3)^˙^+^ spectrum and 20% of the difference spectrum between FAD˙^–^ and FAD to DADS-2 should result in the pure TrpH_420_^(1)^˙^+^ spectrum, since this DADS resembles the decay of [FAD˙^–^,TrpH_420_^(1)^˙^+^] into [FAD˙^–^,TrpH^(2/2–3)^˙^+^] and ground state species. Considering the protein environment which potentially allows for specific interactions with TrpH˙^+^, the global shapes of the obtained species associated spectra (SAS; Fig. 2e and Fig. S14, ESI†) are in good agreement with literature data from solution spectra,^8^,^43^,^44^ and result in concentration–time profiles that follow the discussed models with 20% RPR of [FAD˙^–^,TrpH_420_^(1)^˙^+^] (Fig. 2f and 6). The additional absorption band at *ca.* 410 nm, the broadening of the main absorption band, and significantly enhanced extinction coefficients of the tryptophanyl radical cation spectra, compared to published data from solution spectra,^44^ might inform on specific electrostatic interactions within the protein pocket. These interesting findings will be addressed in future theoretical investigations, which are out of the scope of the present work.

### Proton release of terminal tryptophanyl radical cation

Following the kinetics on the ps time scale, only decaying or constant signals are observed on the μs timescale. The broad TA, that arises within the pulsed excitation (Fig. 3a) may be assigned to the terminal RP, [FAD˙^–^,TrpH_394_^(4)^˙^+^], since it is similar but not identical to the TA spectra at the end of the ps TA data giving further evidence that the terminal RP [FAD˙^–^,TrpH_394_^(4)^˙^+^] is not formed within the first 3 ns but rather between >3 ns and <100 ns as estimated from our resolution limit (*ca.* 100 ns). The TA spectrum decays on the red shoulder within 3 μs while the other parts of the TA stay constant over the time range of 10 μs. This is also reflected in the global fit result, that shows two DADS with rate constants of (1 μs)^–1^ and (≫10 μs)^–1^ (Fig. 3b). The first DADS is almost entirely positive which also indicates no rising signal from a newly formed species. Furthermore, the missing ground state bleach of FAD in DADS-1 also indicates no recombination reaction (Fig. S15, ESI†). Since the two observed rate constants differ by a factor ≫10, the DADS represent the difference between the precursor and formed product state. The intuitive scenario explaining the data may be the proton release of TrpH_394_^(4)^˙^+^ forming its neutral form Trp_394_^(4)^˙ consistent with a previous report (to note in this work the deprotonation was recorded at 6 °C and therefore, the lifetime is 2.5 times slower).^26^ According to the DADS the extinction coefficients of the created species must be ≤ than the extinction coefficients of its precursor state in the entire spectral range. The second DADS does not show any features of a TrpH˙^+^ (neither a band nor a shoulder indicating TrpH˙^+^ contributions is observed), which indicates a complete proton transfer (PT), and thus, DADS-2 accounts for the RP [FAD˙^–^,Trp_394_^(4)^˙] minus ground state contribution (Fig. 3b and Fig. S15, ESI†). By subtracting the difference spectrum between FAD˙^–^ and FAD from DADS-2 such that all flavin spectral features disappear (orange line in Fig. 3c and Fig. S16, ESI†), this results in the pure Trp_394_^(4)^˙ spectrum in good agreement in terms of shape with the known solution spectrum (orange dashed line in Fig. 3c). Addition of the resulting Trp_394_^(4)^˙ spectrum to DADS-1 should result in the pure TrpH_394_^(4)^˙^+^ spectrum. Consistent with our ps TA data the obtained TrpH_394_^(4)^˙^+^ spectrum is broader compared to the known solution spectrum for this species (blue lines in Fig. 3c).^44^ These SAS allow for a decomposition of the raw data into concentration–time profiles following the law of conserved mole fraction (Fig. 3d).

### Blue-light illumination results in the loss of FAD binding

**Fig. 4:**
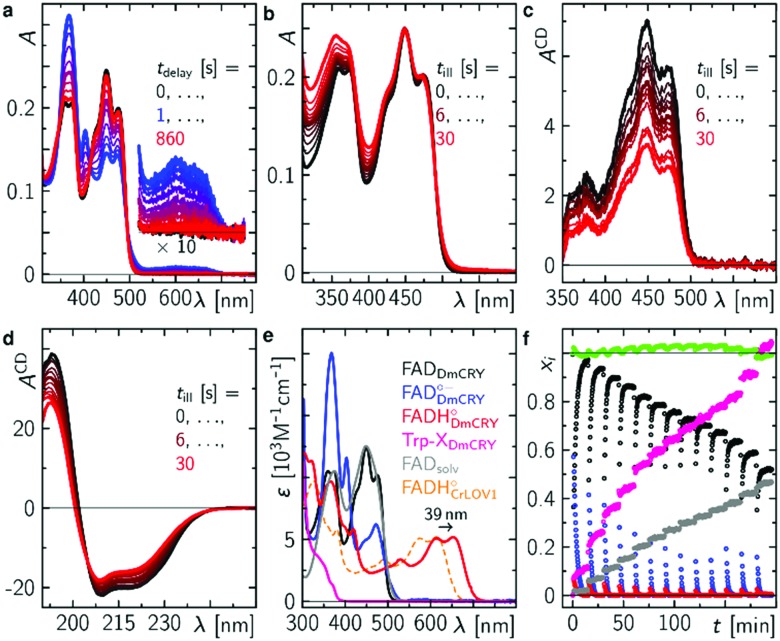
Final events after photo excitation of FAD inside DmCRY forming an irreversible Trp photo-product. (a) Sequence of absorption spectra of a freshly in the dark purified sample of DmCRY following a 1 s blue light pulse. This experiment was performed 13 × (10 × 1 s, 2 × 5 s and 1 × 10 s blue light pulse). (b) Absorption spectra after illumination as indicated and full re-oxidation (see for instance first re-oxidation sequence after first light pulse in panel (a)) and corresponding CD absorption spectra in the Vis (c) and UV (d) spectral range of the identical sample in (a) and (b). (e) Basis spectra for data decomposition: FAD_DmCRY_ dark state spectrum from freshly purified sample in the dark, FAD_DmCRY_˙^–^ from ref. 8, FADH_DmCRY_˙ is the 39 nm redshifted spectrum of FADH_CrLOV1_˙ from ref. 33, Trp-X_DmCRY_ is the final spectrum of the entire sequence minus a linear combination of FAD_solv_, FAD_DmCRY_, a scatter function, and an additional correction for small oscillatory features due to loss in fine structure signatures of the FAD_DmCRY_ spectrum (Fig. S17, ESI†), FAD_solv_ recorded in this work. The spectrum of Trp-X_DmCRY_ is arbitrarily scaled to the total amount of FAD_DmCRY_ used in the decomposition. (f) Mole fraction *vs.* experimental time profiles resulting from data decomposition using the basis spectra in e.

Finally, we studied the photocycle of DmCRY by stationary UV-Vis and CD spectroscopies under aerobic conditions and in the absence of reducing agents. The absorption spectrum of ground state oxidised FAD of an in darkness purified DmCRY sample shows characteristic vibrational fine structural features around 350 and 450 nm, typically observed for protein-bound FAD (black line in Fig. 4a). Illumination with a 1 s blue-light pulse leads to the formation of the stable FAD˙^–^ with its typical absorption maxima at 472, 403, and 367 nm and weak but distinct absorption between 550 and 700 nm due to eT along the Trp tetrad to the isoalloxazine moiety as shown above (blue line in Fig. 4a). The shape of the small absorption band between 550 and 700 nm agrees well with published SAS of flavin neutral radical^33^ but is redshifted by 39 nm. A similar redshift was previously observed for FADH˙ in type II cryptochromes.^8^ Thus, in contrast to previous reports in which formation of the weak broad band between 550 and 700 nm was assigned to the FAD˙^–^,^28^ we assign this band to FADH˙. Based on the absorption ratio of FAD˙^–^ and FADH˙ and the p*K*_a_(FAD˙^–^/FADH˙)^45^ = 8 the local pH of the FAD binding pocket can be estimated to 9.0. Subsequent incubation of the sample in darkness leads to re-oxidation of FAD˙^–^ back to FAD. However, as evident small but distinct deviations in the spectra obtained before irradiation (black) and after re-oxidation (red) at *ca.* 320, 360–370, 400, 450, and 475 nm are observed, which do not disappear even when the sample is incubated in darkness for several hours. Further sequences of pulsed illumination followed by full re-oxidation show increased absorption at <400 nm accompanied by the loss of the characteristic vibrational fine structure of bound FAD indicating the release of FAD from the FAD binding pocket (Fig. 4b). The loss of flavin binding is confirmed by circular dichroism (CD) in the visible region. In contrast to free FAD in solution, where negative and smaller signals are observed, the protein bound FAD gives positive contributions due to the chiral environment of the protein pocket.^8^ The positive contributions decrease with increasing number of light pulses (Fig. 4c). CD spectra recorded in the ultraviolet region (Fig. 4d) show that DmCRY undergoes light-induced conformational changes consistent with previous reports.^7^ The increased absorption at <400 nm is assigned to a new product that has not been reported so far. Its absorption spectrum can be obtained by subtracting spectral contributions of (un)bound FAD from the last spectrum of the sequence (Fig. 4e and Fig. S17, ESI†). The formation of a similar product spectrum is also observed in the flavin photocatalysis of tryptophan (Fig. S18, ESI†). Therefore, the observed new species can be assigned to a yet unidentified Trp product, Trp-X. This assignment is further confirmed by the observation of a similar spectrum of a photoproduct of a Trp-containing peptide.^46^

Taking all contributing species spectra, the data can be decomposed into physically meaningful concentration–time profiles (Fig. 4e and f). As bound FAD decreases to *ca.* 50% unbound FAD increases accordingly and less flavin radicals are formed. Simultaneously, Trp-X increases with the number of illumination pulses.

**Fig. 5:**
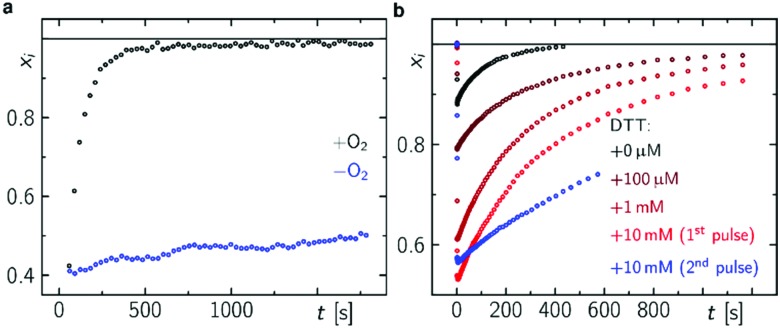
FAD re-oxidation in DmCRY in dependence of O_2_ and DTT. (a) Mole fraction *vs.* time profiles after 1 s blue light pulse in dependence of O_2_ (a) and after 100 ms blue light pulse in dependence of DTT concentration (b) as indicated.

**Fig. 6:**
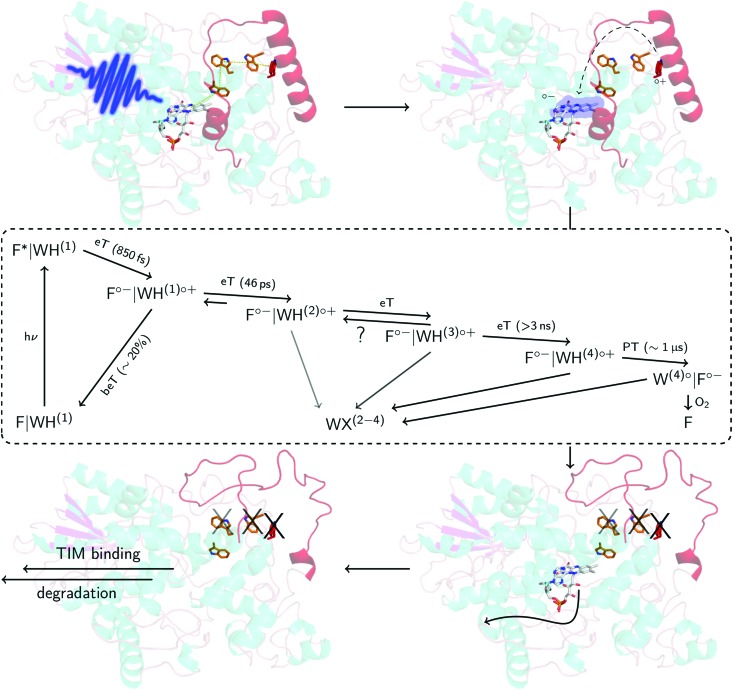
Proposed mechanism in DmCRY at a molecular level. Cartoon representation of DmCRY crystal structure (pdb code: 4GU5)^14^ with stick representations of FAD (grey carbons) and the adjacent conserved tryptophan tetrad (orange/red carbons). Following the arrows: (1) photo-excitation of FAD; (2) eT along Trp-tetrad to FAD with details in dashed box derived from this study; (3) unwinding of CTT, Trp decomposition, and FAD release; (4) TIM binding and final degradation.

In accordance with previous reports,^29^,^30^ molecular oxygen is the main cause for FAD˙^–^ re-oxidation (Fig. 5a). In the presence of reducing agents, such as di-thiothreitol (DTT), bound FAD is bleached stronger with increasing DTT concentration due to eT from DTT to FAD. DTT is small enough to enter the FAD binding pocket similar to the situation with experiments with external addition of thiols to LOV domains^33^ (Fig. S19, ESI†). As a further consequence the formed DTT radicals, DTT˙, consume the molecular oxygen^47^ so that the FAD˙^–^ re-oxidation becomes slower with each excitation cycle (Fig. 5b). Furthermore, the Trp decomposition product is still observed in the presence of DTT excluding the re-reduction of tryptophanyl radical. The Trp decomposition can also be observed in previously presented data, *e.g.*ref. 28 and 49. As a consequence, no RPR occurs for the terminal RP, *i.e.* [FAD˙^–^,Trp_394_^(4)^˙]. This is further confirmed by TA in the ms to s time range, where Trp˙ decays on a ms timescale (*τ* = 187 ms) while FAD˙^–^ decays on a min timescale in dependence on O_2_ concentration (Fig. 5a and Fig. S20, ESI†). Trp_394_^(4)^˙ will potentially decompose *via* H_2_O reaction, since FAD is re-generated *via* a different pathway. Thus, once DmCRY undergoes a photoreaction it ends up as a different ‘molecule’, *i.e.* DmCRY-Trp(4,decomposed). Accordingly, another identical photoreaction of the new DmCRY-Trp(4,decomposed) is impossible. However, the DmCRY-Trp(4,decomposed) might be able to undergo another photoreaction, but this time a different terminal RP would be formed, *i.e.* [FAD˙^–^,Trp_342_^(3)^˙]. This sequence will potentially end with DmCRY-Trp(2,decomposed), since RPR between FAD˙^–^ and Trp_420_^(1)^˙ is expected to be fast due to their close proximity (Fig. 6). With accumulation of decomposed Trps in DmCRY the chance for FAD release enhances so that several photons are required. Hence a low quantum yield for FAD release is expected, which is in line with our data (Fig. 4f) as a reasonable estimate for the product quantum yields of 80% for FAD˙^–^ and 6% for free FAD shows (see ESI† for product quantum yield estimation). *In vivo* studies suggest that a single excitation pulse (1 ms) is sufficient to drive 80% of DmCRY degradation in 1 h.^49^ Thus, it is tempting to speculate that with increasing amount of decomposed Trps in DmCRY the *in vivo* degradation will be accelerated.

## Conclusions

Here, we have investigated in detail the initial photochemical events in the blue-light photoreceptor DmCRY. We resolved the first eT followed by a second eT that results either in the pure TrpH_397_^(2)^˙^+^ or the formation of an equilibrium between TrpH_397_^(2)^˙^+^ and TrpH_342_^(3)^˙^+^ along the conserved tryptophan tetrad and the final deprotonation of the terminal tryptophanyl radical cation. The terminal RP, [FAD˙^–^,TrpH_394_^(4)^˙^+^], is estimated to be formed between >3 ns and <100 ns. On a longer timescale, we observe FAD release from the DmCRY FAD-binding pocket and Trp decomposition in a yet unidentified Trp-X product, thus providing further insights into the DmCRY reaction mechanism. Based on our results, we propose that light is a negative regulator of DmCRY stability even under *in vitro* conditions where the proteasomal machinery is missing that is in line with its biological function, *i.e.* entrainment of the circadian clock.^48^ Consistent with previous reports, absorption of a photon leads to reduction of the protein-bound FAD *via* consecutive eT along the Trp-tetrad until the terminal surface exposed TrpH˙^+^ radical is formed. We suggest that the formation of positively charged TrpH˙^+^ radicals during eT leads to electrostatic repulsions with the nearby positively charged amino acids of the protein backbone resulting in global protein conformational changes which is in line with our MD simulation on all four potential radical pairs (compare the different orientations of the tryptophanyl radicals in the ‘dark’ ground and ‘light’ radical pair states in Fig. S11, S12, and Table S1, ESI†). Furthermore, we hypothesize, consistent with the suggestion that generated charges drive conformational changes,^52^ that the positive charge of the terminal TrpH˙^+^ will cause profound conformational changes in helix α22 that is directly connected to the CTT resulting in its release from the FAD-binding pocket (Fig. 6 and Fig. S21, ESI†) accompanied by Trp decomposition and allowing FAD release. Consistent with our suggestion, in previous studies it was shown that absorption of light by DmCRY leads to conformational changes in the CTT, that were shown to be largely irreversible,^49^–^51^ thus allowing the interaction with TIM followed by proteasomal degradation of both proteins. Further, we suggest that similar to animal type II CRY,^53^ the E3 ubiquitin ligase, Ramshackle,^13^ binds to the DmCRY FAD-binding pocket making the FAD release a requirement for subsequent ubiquitylation and its proteasomal degradation, thus, allowing the next cycle of the circadian clock (Fig. 6 and Fig. S22, ESI†). In conclusion, our work provides profound insights into the DmCRY mechanism. In contrast to the present understanding, DmCRY does not undergo a photocycle but rather an irreversible inactivation reaction. Furthermore, we provide the link between the initial photochemistry of DmCRY and the subsequent dark processes leading to signal transduction on a molecular level.

## Conflicts of interest

There are no conflicts to declare.

## Supplementary Material

Supplementary information
