# Supplementary material for: The sacrificial inactivation of the blue-light photosensor cryptochrome from Drosophila melanogaster
Source: Phys Chem Chem Phys. 2018 Nov 12;20(45):28767–76. doi: 10.1039/c8cp04671a (PMC6250122; doi:10.1039/c8cp04671a)
Supplement: Supplementary file 1 [file CP-020-C8CP04671A-s001.pdf]

Electronic Supplementary Material (ESI) for Physical Chemistry Chemical Physics.
This journal is © the Owner Societies 2018

Supplementary Information

The sacrificial inactivation of the blue-light photosensor Cryptochrome from *Drosophila melanogaster*

Roger J. Kutta,[1,2*] Nataliya Archipowa,[1] and Nigel S. Scrutton[1*]

1. Manchester Institute of Biotechnology (MIB) and School of Chemistry, The University of Manchester, 131 Princess Street, Manchester, M1 7DN, UK.

2. Institut für Physikalische und Theoretische Chemie, Universität Regensburg, Universitätsstr. 31, 93053 Regensburg, Germany.

Supplementary Text

Theory of time-dependent absorption depolarisation

The dimensionless anisotropy for a given transient absorption band is defined as

$$r(t,\lambda) = \frac{\Delta A_{\parallel}(t,\lambda) - \Delta A_{\perp}(t,\lambda)}{\Delta A_{\mathrm{T}}(t,\lambda)} = \frac{\Delta A_{\parallel}(t,\lambda) - \Delta A_{\perp}(t,\lambda)}{\Delta A_{\parallel}(t,\lambda) + 2\Delta A_{\perp}(t,\lambda)} \quad (1)$$

where $\Delta A_{\parallel}(t,\lambda)$ and $\Delta A_{\perp}(t,\lambda)$ are the difference absorptions probed parallel and perpendicular to the excitation polarisation, respectively. $\Delta A_{\mathrm{T}}(t,\lambda)$ is the total amount of difference absorption along all three space coordinates, so that the perpendicular contribution needs to be counted twice.

In none rigid media, *e.g.* liquid solution, rotational diffusion is the main cause of the disappearance of the anisotropy induced by polarised excitation. For a particular absorption transition of a spherical molecule, the decay of anisotropy is given by Jabłoński's equation[1], eqn. (2).

$$r(t) = r_0 \exp(-k_\theta t), \quad (2)$$

where r_0 is the anisotropy at $t = 0$ given by eqn. (3).

$$r_0 = \frac{2}{5}\left(\frac{3\cos^2(\beta) - 1}{2}\right) \quad (3)$$

k_θ is the rate constant of rotational correlation, and β is the angle between the excited transition and the probed transition moments. The rate constant of rotational correlation is related to the rotational diffusion coefficient *via* $k_\theta = 6D$. Eqn. (4) gives the reverse relation between β and the anisotropy r_0

$$\beta = \cos^{-1}\left(\sqrt{\frac{5r_0 + 1}{3}}\right) \quad (4)$$

Thus, the extreme values for a single contributing molecule of the anisotropy are 0.4 ($\beta = 0°$) in case of parallel transition moments and −0.2 ($\beta = 90°$) in case of orthogonal transition moments. The anisotropy is lost at all times for $\alpha = 54.74°$. Under these conditions the observed kinetics and dynamics represent entirely state specific characteristics and no contributions due to rotational diffusion.

In case of a multicomponent system, *i.e.* several species contribute to a particular absorption band in the time-resolved absorption experiment, the total anisotropy $r^{\mathrm{total}}(t)$ is given as the

sum of all N individual anisotropies arising from each individual species weighted by the fractional contribution to the observed absorption signal according to eqn. (5)

$$r^{\text{total}}(\lambda,\text{t}) = \sum_{i=1}^{N} r_i(\lambda,t)\frac{\Delta A_i(\lambda,t)}{\Delta\text{A}(\lambda,\text{t})} \quad (5)$$

From eqn. (5) it becomes directly evident that for either purely positive or purely negative absorption signals the limits for $r^{\text{total}}(\lambda,t)$ are also restricted to 0.4 and −0.2, as for each individual contribution, since the fraction of contributors is always ≤ 1. However, when positive and negative absorption signals contribute simultaneously, *i.e.* ground state bleach or stimulated emission plus excited state or transient absorption, then these limits no longer hold and the anisotropy can reach values beyond since in this case the total absorption signal is no longer a pure sum of absolute contributions but rather includes subtraction terms.

In terms of rotational diffusion of a globular protein, one can consider an Einstein sphere. Using Einstein's law the rate constant of rotational correlation is given in eqn. (6)

$$(k_\theta)^{-1} = (6D)^{-1} = \tau_\theta = \frac{\eta V_\text{m}}{RT} \approx \frac{\eta M}{RT}(\bar{v}+h), \quad (6)$$

where η is the viscosity (in kg(dm)$^{-1}$s^{-1}), T is the temperature (in °K), R is the gas constant (in kg(dm)^{2}s^{-2}mol^{-1}K^{-1}), V_m is the volume of one mole of the rotating unit (in (dm)^{3}mol^{-1}), M is the molecular mass of the rotating unit (in mol), $\bar{v}$ is the specific volume of the rotating unit (in (dm)^{3}g^{-1}), and h is the hydration of the rotating unit (in (dm)^{3}g^{-1}). A typical value of $\bar{v}$ for proteins is close to 0.73 mL g^{-1}. Typically, h can be estimated *via* the observation that 1 g of protein binds *ca.* 0.23 g H_2O[2]. Thus, for DmCRY with a molecular mass of 66716 g mol^{-1} and the used buffer conditions, *i.e.* 10% glycerol in aqueous buffer yielding a viscosity of *ca.* 0.0122 kg (dm)$^{-1}$ s^{-1},[3,4] one can estimate a rotational correlation rate constant of *ca.* (32 ns)$^{-1}$. Since total depolarisation is reached in *ca.* 5 times the rotational correlation lifetime, this shows that within the time window of 3 ns of the time-resolved absorption experiment, DmCRY will depolarise to a negligible value of *ca.* 1.9%. Then, based on the crystal structure of DmCRY (pdb code: 4GU5)[5] the expected anisotropies r_0 of individual contributions to certain absorption bands arising from the blue-light induced formation of the four potential radical pairs, *i.e.* [FAD$^{\circ-}$,$\text{Trp}_{420}^{(1)\,\circ+}$], [FAD$^{\circ-}$, $\text{Trp}_{397}^{(2)\,\circ+}$], [FAD$^{\circ-}$, $\text{Trp}_{342}^{(3)\,\circ+}$], and [FAD$^{\circ-}$, $\text{Trp}_{394}^{(4)\,\circ+}$], with either a protonated or deprotonated tryptophan radical can be estimated in first approximation. The transition dipole moments of the most prominent absorption bands of all potentially contributing radical species, *i.e.* ^{1}FAD, ^{1}FAD*, ^{2}FAD$^{\circ-}$,

$^2Trp^{\circ+}$, $^2Trp^{\circ}$, $^2Tyr^{\circ+}$, and $^2Tyr^{\circ}$, were calculated quantum chemically (see section Materials and Methods for more details) and from the orientations given by the crystal structure the corresponding anisotropies were evaluated. However, recently we have shown that the crystal structure might not resemble the energetic minimum on the potential energy surface[6] potentially due to artificial packing effects and since proteins are in general 'dynamic' structures, we performed molecular dynamics simulations on DmCRY in its 'dark' ground state and in its four 'light' radical pair states in order to account for a more realistic relaxed and dynamic structure (see section Materials and Methods for more details). These are summarized in Tab. S1 & S2 and Fig. S1. As can been seen, DmCRY shows high flexibility in the trajectories over 100 ns and that sampling along 100 ns might not cover the complete configuration space of DmCRY. Thus, we performed 5 individual runs over 100 ns and averaged over all configurations found, in order to obtain a reasonable estimate for the solution structure as can be seen in the small deviations in the averaged trajectories (red lines in Fig. S11-13). Since DmCRY shows high flexibility, these fluctuations need to be taken into account in the determination of the expected theoretical anisotropies. The static picture from the crystal structures obviously cannot account for this, thus, our MD data give a more realistic estimate for the expected anisotropies.

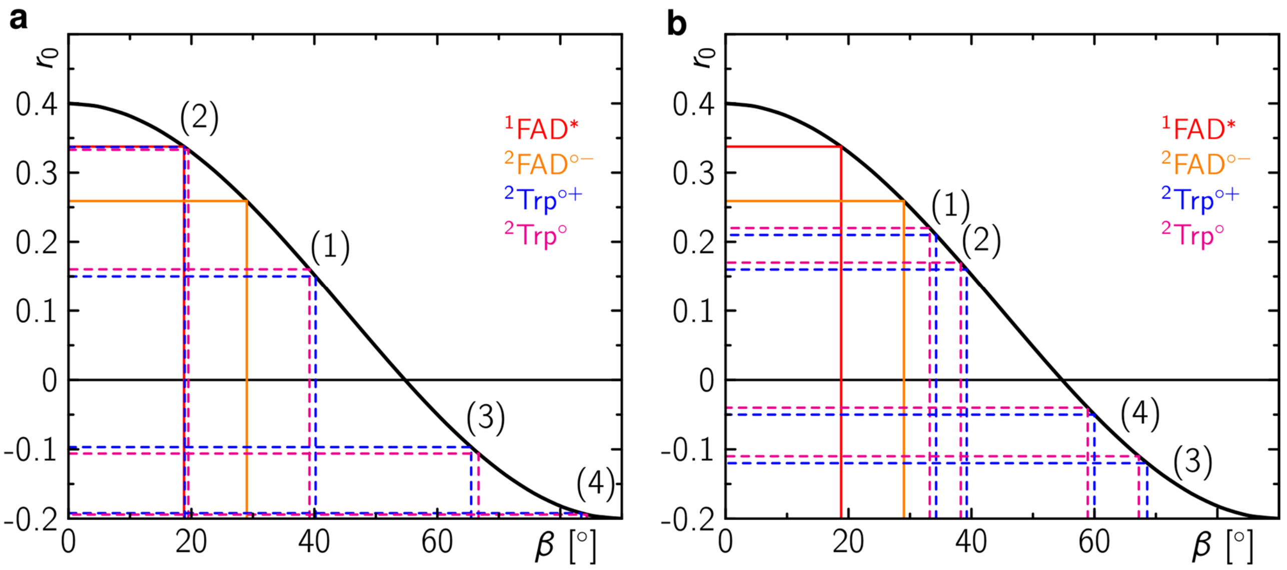

Fig. S1 Expected anisotropies and corresponding angles between the transition moment for flavin excitation, μ_{01}, to the transition moments of the most prominent transition dipole moments of all potentially contributing species of the eT pathway based on the DmCRY crystal structure (a; pdb code: 4GU5)[5] and based on relaxed structures from MD simulations (b). The black line is a plot of eqn. (3).

residue X	transition	E_{ij}	$\measuredangle(\mu_{01}^{\mathrm{FAD}}, \mu_{ij}^{\mathrm{X}})$	r_0	$\langle r_0 \rangle_{\mathrm{dark}}^{\mathrm{MD}}$	$\langle r_0 \rangle_{\mathrm{light}}^{\mathrm{MD}}$
^{1}FAD*	3←1	1.94/638	41.4	0.14	0.14	0.14
	4←1	2.03/610	59.6	−0.05	−0.05	−0.05
	**5←1**	**2.39/520**	**2.7**	**0.40**	**0.40**	**0.40**
	**6←1**	**2.91/426**	**18.8**	**0.34**	**0.34**	**0.34**
^{1}FAD$^{\circ -}$	1←0	1.83/678	44.4	0.11	0.11	0.11
	2←0	2.40/516	25.4	0.29	0.29	0.29
	**3←0**	**2.51/494**	**15.0**	**0.36**	**0.36**	**0.36**
	**4←0**	**3.27/379**	**29.0**	**0.26**	**0.26**	**0.26**
$\mathrm{Trp}_{420}^{(1)\,\circ}$	**2←0**	**2.63/471**	**39.2**	**0.16**	**0.21**	**−0.14**
	3←0	3.65/339	48.5	0.06		
	4←0	4.10/303	41.5	0.14		
$\mathrm{Trp}_{420}^{(1)\,\circ +}$	**2←0**	**2.11/588**	**40.2**	**0.15**	**0.21**	**−0.07**
	3←0	3.54/350	46.6	0.08		
	4←0	3.83/324	18.5	0.34		
$\mathrm{Trp}_{397}^{(2)\,\circ}$	**2←0**	**2.63/471**	**18.9**	**0.34**	**0.16**	**−0.01**
	3←0	3.65/339	28.4	0.27		
	4←0	4.10/303	59.7	−0.05		
$\mathrm{Trp}_{397}^{(2)\,\circ +}$	**2←0**	**2.11/588**	**19.5**	**0.33**	**0.16**	**0.05**
	3←0	3.54/350	26.2	0.28		
	4←0	3.83/324	34.5	0.21		
$\mathrm{Trp}_{342}^{(3)\,\circ}$	**2←0**	**2.63/471**	**65.5**	**−0.10**	**−0.12**	**0.10**
	3←0	3.65/339	72.7	−0.15		
	4←0	4.10/303	44.2	0.11		
$\mathrm{Trp}_{342}^{(3)\,\circ +}$	**2←0**	**2.11/588**	**66.7**	**−0.11**	**−0.12**	**−0.11**
	3←0	3.54/350	71.6	−0.14		
	4←0	3.83/324	40.2	0.15		
$\mathrm{Trp}_{394}^{(4)\,\circ}$	**2←0**	**2.63/471**	**83.4**	**−0.19**	**−0.05**	**−0.16**
	3←0	3.65/339	82.9	−0.19		
	4←0	4.10/303	85.5	−0.20		
$\mathrm{Trp}_{394}^{(4)\,\circ +}$	**2←0**	**2.11/588**	**84.3**	**−0.19**	**−0.05**	**−0.15**
	3←0	3.54/350	83.7	−0.19		
	4←0	3.83/324	88.9	−0.20		

Tab. S1 Expected anisotropies and corresponding angles between the transition moment for flavin excitation, μ_{01}, to the transition moments of the most prominent transition dipole moments of all potentially contributing species of the eT pathway based on the DmCRY crystal structure (pdb code: 4GU5)[5] and based on relaxed structures from MD simulations.

Residue X	transition	E_{ij} [eV/nm]	$\measuredangle(\mu_{01}^{\text{FAD}}, \mu_{ij}^{\text{X}})$ [°]	r_0	$\langle r_0 \rangle_{\text{dark}}^{\text{MD}}$
$\text{Tyr}_{158}^{(1)\circ}$	**2←0**	**3.21/386**	**54.8**	**0.00**	**−0.04**
	3←0	4.31/288	53.6	0.01	
$\text{Tyr}_{158}^{(1)\circ+}$	**2←0**	**3.11/399**	**57.1**	**−0.02**	**−0.06**
	3←0	4.53/274	54.9	−0.00	
$\text{Tyr}_{317}^{(2)\circ}$	**2←0**	**3.21/386**	**27.9**	**0.27**	**0.23**
	3←0	4.31/288	73.1	−0.15	
$\text{Tyr}_{317}^{(2)\circ+}$	**2←0**	**3.11/399**	**26.7**	**0.28**	**0.24**
	3←0	4.53/274	27.8	0.27	
$\text{Tyr}_{319}^{(3)\circ}$	**2←0**	**3.21/386**	**64.0**	**−0.09**	**0.02**
	3←0	4.31/288	67.7	−0.11	
$\text{Tyr}_{319}^{(3)\circ+}$	**2←0**	**3.11/399**	**65.4**	**−0.10**	**0.02**
	3←0	4.53/274	64.1	−0.09	

Tab. S2 Expected anisotropies and corresponding angles between the transition moment for flavin excitation, μ_{01}, to the transition moments of the most prominent transition dipole moments of the secondary/alternative eT pathway, *i.e.* $^2\text{Tyr}^{\circ+}$, and $^2\text{Tyr}^{\circ}$, based on the DmCRY crystal structure (pdb code: 4GU5)[5] and based on relaxed structures from MD simulations.

Estimation of product quantum yields

FAD release requires unfolding of the peptide following Trp decomposition. The chance for FAD release should increase with the number of decomposed Trp's, since this will eventually cause destabilisation of the secondary/tertiary protein structure. However, with increasing number of decomposed Trp's the probability of RPR increases (due to decreasing distances within the corresponding RPs) so that the chance for further Trp decomposition decreases limiting the rate of FAD release. Thus, the total yield of free FAD is expected to be low, since several subsequent photo-reactions are required for a high probability of FAD release, *i.e.* three photo-reactions (no photo-cycle). According to our time-resolved data, the QY of $FAD^{\circ-}$ should be *ca.* 80% for intact DmCRY, since we have 20% loss due to RPR in the 1st RP. In general, an exact determination of a quantum yield is difficult *per se*. However, we can make a reasonable estimate for the QY for $FAD^{\circ-}$ *via* the following formula:

$$\Phi(\mathrm{FAD}^{\circ-}) = \frac{c(\mathrm{FAD}^{\circ-}) \cdot V_{\mathrm{sample}}}{P_{\mathrm{LED}} \cdot \lambda_{\mathrm{exc}} \cdot \Delta t \cdot f_{\mathrm{geo}} \cdot f_{\mathrm{abs}}} N_{\mathrm{A}} \cdot c \cdot h \qquad (7)$$

where:

$c(\mathrm{FAD}^{\circ-})$:= concentration of formed $FAD^{\circ-}$ (12 μM)

V_{sample} := sample volume (120 μL)

P_{LED} := total LED power (20 mW)

λ_{exc} := excitation wavelength (455 nm)

Δt := pulse width of LED (1 s)

f_{geo} := geometric factor that accounts for the actual illuminated sample area (*ca.* 22% of the homogenous LED spot hits the sample)

f_{abs} := factor that accounts for absorbed photons based on absorption (OD^{455nm} = 0.25) and pathlength (2 mm) of the sample (*ca.* 11%)

N_{A} := Avogadro's constant

c := speed of light in vacuum

h := Planck's constant

This gives a QY for $FAD^{\circ-}$ of ~80% which is in line with our time-resolved data. In a similar way the QY for FAD release (using $c(\mathrm{free\ FAD})$ = 1 μM) is given to ~6% confirming our expected low QY of FAD release.

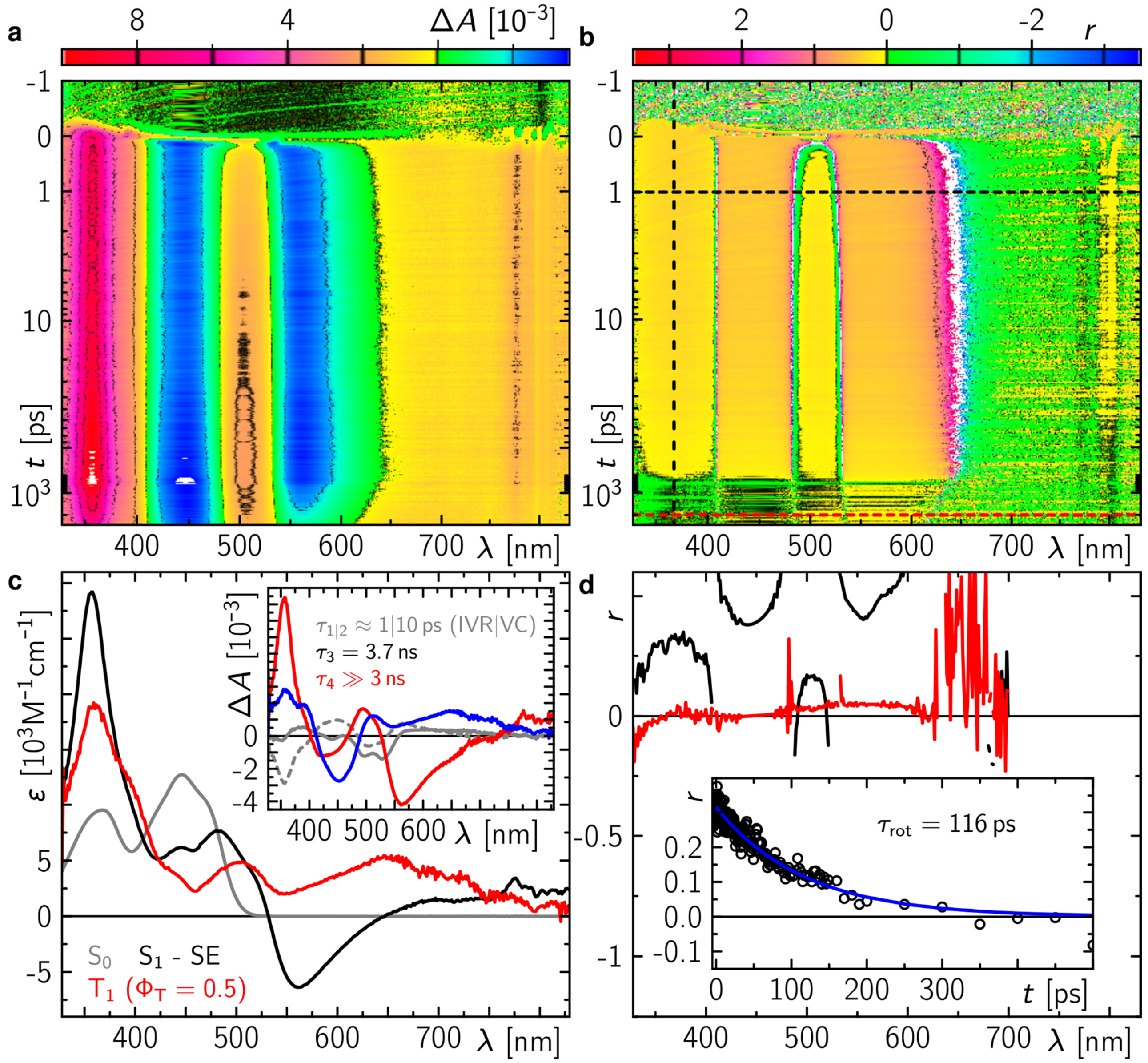

Fig. S2 Transient absorption and anisotropy of free flavin. Tetraacetylriboflavin dissolved in ACN/H_2O (25 Vol%/75 Vol%) was excited into its $S_1 \leftarrow S_0$ transition at 450 nm. **a**: Pump and probe set to magic angle. **b**: Pump and probe set to either 0° or 90° resulting in anisotropy data according to eqn. (1). **c**: Species associated spectra in agreement to standard photo-physical model including S_1 (note that the negative band peaking at *ca.* 560 nm originates from the stimulated emission (SE) of flavin) and T_1 states. Inset shows decay associated difference spectra from global fit with four exponentials. The two grey spectra indicate the spectral dynamics of internal vibrational relaxation (IVR, solid grey line) and vibrational cooling (VC, dashed grey line) with typical lifetimes for those processes. **d**: Spectral slices along the data in **b** as indicated by horizontal dashed lines. Inset shows the anisotropy decay as indicated by the vertical dashed line in **b** following the typical rotational correlation lifetime observed for flavin.

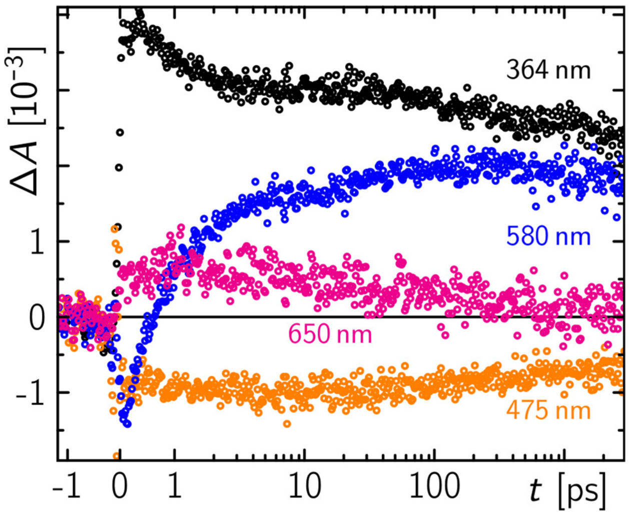

Fig. S3 Initial events after photo excitation of FAD inside DmCRY. Shown are selected time traces of the transient absorption data matrix from Fig. 2**a** as indicated.

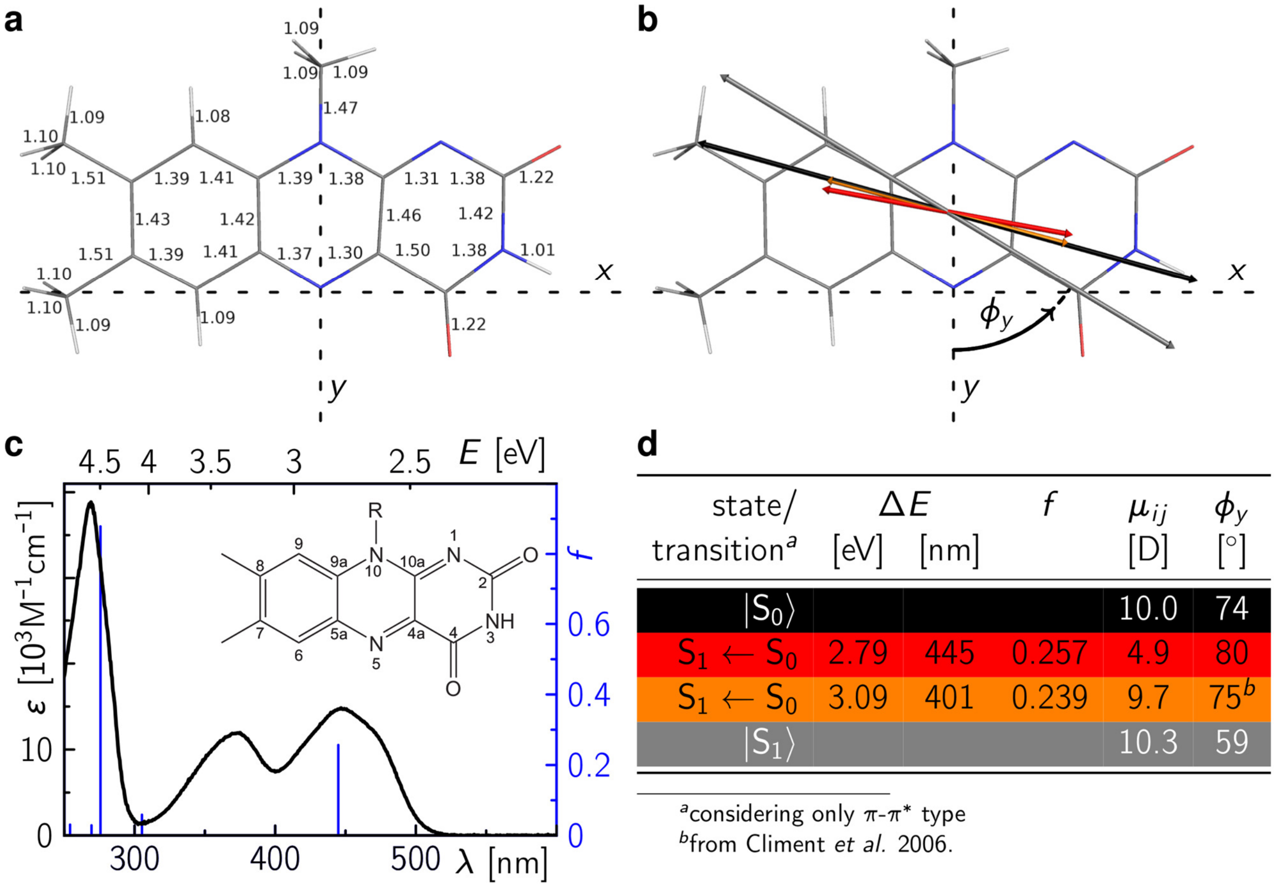

state/ transition[a]	ΔE [eV]	[nm]	f	μ_{ij} [D]	ϕ_y [°]
$\lvert S_0 \rangle$				10.0	74
$S_1 \leftarrow S_0$	2.79	445	0.257	4.9	80
$S_1 \leftarrow S_0$	3.09	401	0.239	9.7	75[b]
$\lvert S_1 \rangle$				10.3	59

[a]considering only π-π^* type
[b]from Climent *et al.* 2006.

Fig. S4 Quantum chemical calculations of flavin ground state. a: Optimised geometry. **b**: Static dipole moment of ground and excited states as well as most prominent transition dipole moments. Colour coding as in **d**. **c**: Experimental (black) and theoretical (blue) absorption spectra. **d**: Details to dipole moments and transition dipole moments shown in **b** (Transition energy, ΔE; oscillator strength, f; static/transition dipole moments, μ_{ij}; angle to y-axis, ϕ_y). Reference to Climent *et al.* 2006.[8]

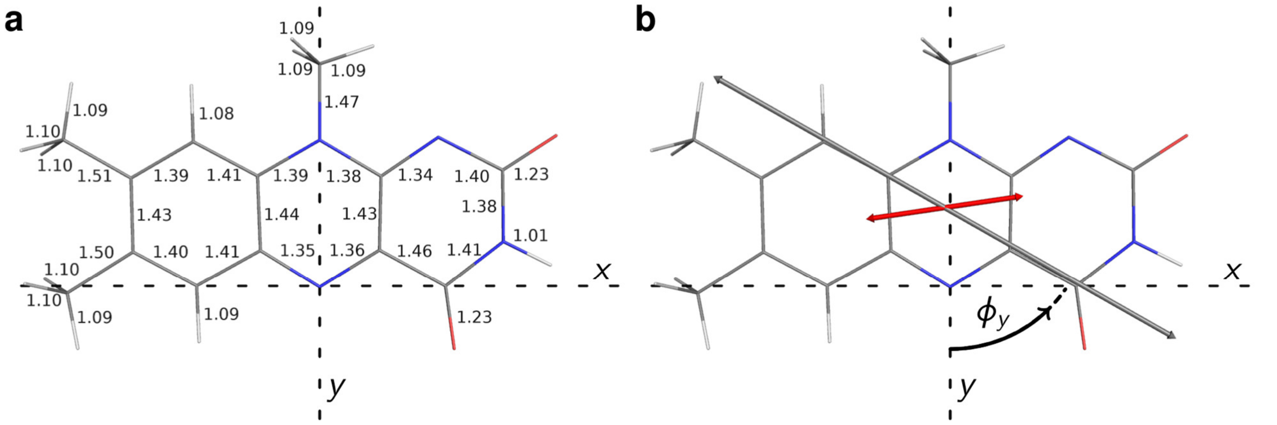

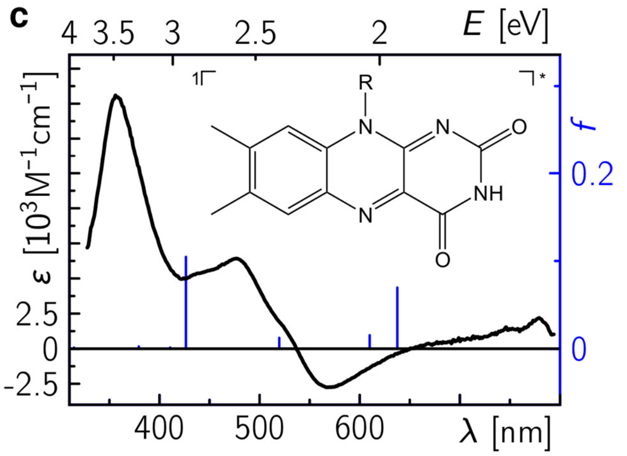

d

state/ transition[a]	ΔE [eV]	ΔE [nm]	f	μ_{ij} [D]	ϕ_y [°]
$\vert S_1\rangle$				10.2	60
$S_3 \leftarrow S_1$	1.94	638	0.070	3.1	121
$S_4 \leftarrow S_1$	2.03	610	0.015	1.4	139
$S_5 \leftarrow S_1$	2.39	520	0.012	1.2	82
$S_6 \leftarrow S_1$	2.91	426	0.105	3.1	98

[a]considering only π-π^* type

Fig. S5 Quantum chemical calculations of flavin first excited state. a: Optimised geometry. **b**: Static dipole moment of excited state as well as most prominent transition dipole moments. Colour coding as in **d**. **c**: Experimental (black line of this work; contains negative contributions form stimulated emission peaking at *ca.* 560 nm) and theoretical (blue) absorption spectra. **d**: Details to dipole moments and transition dipole moments shown in **b** (Transition energy, ΔE; oscillator strength, f; static/transition dipole moments, μ_{ij}; angle to y-axis, ϕ_y).

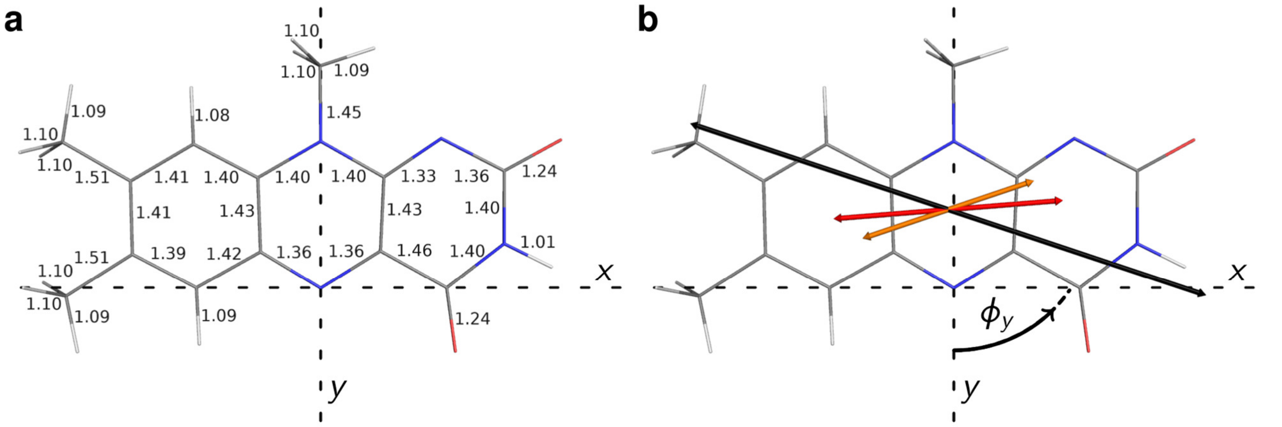

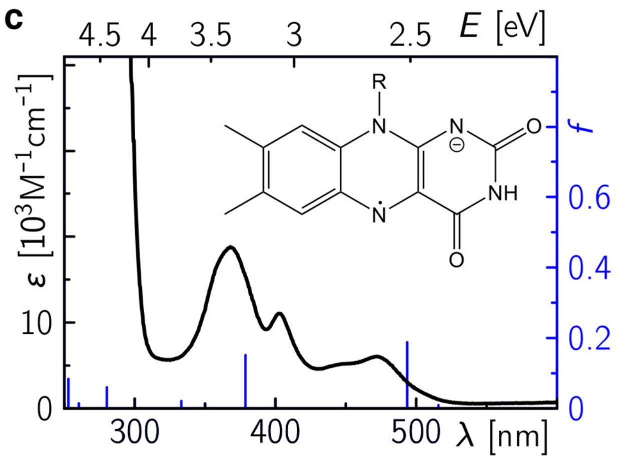

d

state/ transition[a]	ΔE [eV]	[nm]	f	μ_{ij} [D]	ϕ_y [°]
$\|D_0\rangle$				10.5	72
$D_1 \leftarrow D_0$	1.83	678	0.011	1.3	124
$D_2 \leftarrow D_0$	2.40	516	0.010	1.0	105
$D_3 \leftarrow D_0$	2.51	494	0.188	4.4	95
$D_4 \leftarrow D_0$	3.27	379	0.151	3.5	109

[a]considering only π-π^* type

Fig. S6 Quantum chemical calculations of flavin radical anion. a: Optimised geometry. **b**: Static dipole moment of ground state as well as most prominent transition dipole moments. Colour coding as in **d**. **c**: Experimental (black line of this work) and theoretical (blue) absorption spectra. **d**: Details to dipole moments and transition dipole moments shown in **b** (Transition energy, ΔE; oscillator strength, f; static/transition dipole moments, μ_{ij}; angle to y-axis, ϕ_y).

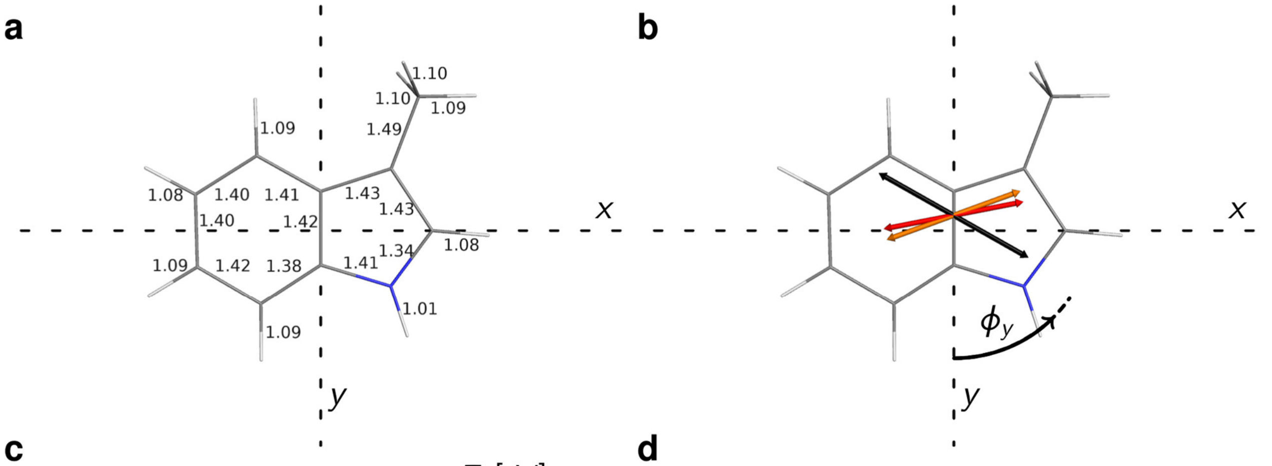

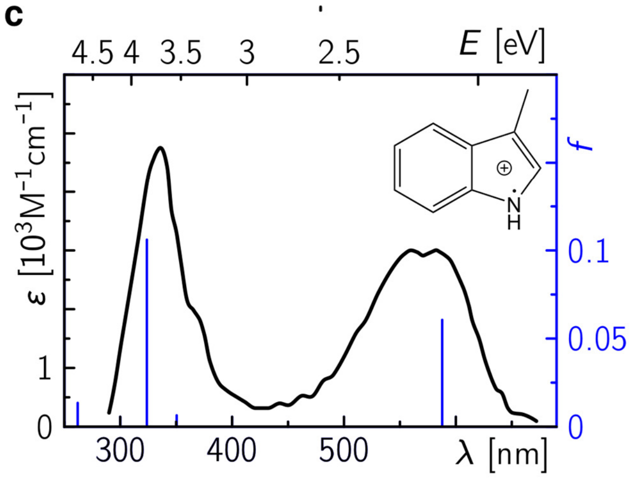

d

state/ transition[a]	ΔE [eV]	[nm]	f	μ_{ij} [D]	ϕ_y [°]
$\lvert D_0 \rangle$				3.3	61
$D_1 \leftarrow D_0$	1.01	1220	10^{-4}	0.2	64
$D_2 \leftarrow D_0$	2.11	588	0.061	2.8	101
$D_2 \leftarrow D_0$					110[b]
$D_3 \leftarrow D_0$	3.54	350	0.006	0.7	108

[a]considering only π-π^* type
[b]from Martin *et al.* 2017.

Fig. S7 Quantum chemical calculations of tryptophan radical cation. a: Optimised geometry. **b**: Static dipole moment of ground state as well as most prominent transition dipole moments. Colour coding as in **d**. **c**: Experimental (black)[9] and theoretical (blue) absorption spectra. **d**: Details to dipole moments and transition dipole moments shown in **b** (Transition energy, ΔE; oscillator strength, f; static/transition dipole moments, μ_{ij}; angle to y-axis, ϕ_y). Reference to Martin *et al.* 2017.[10]

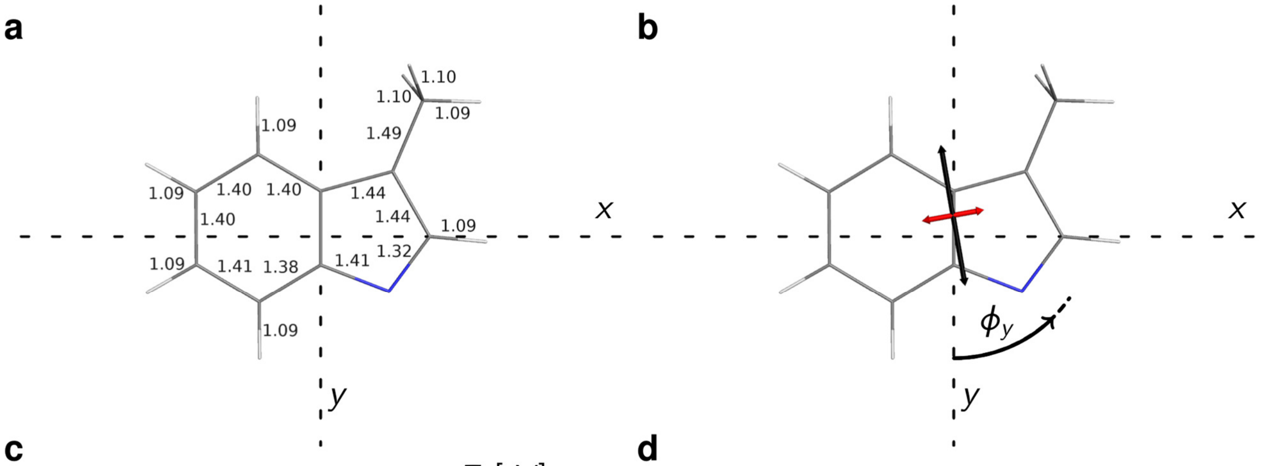

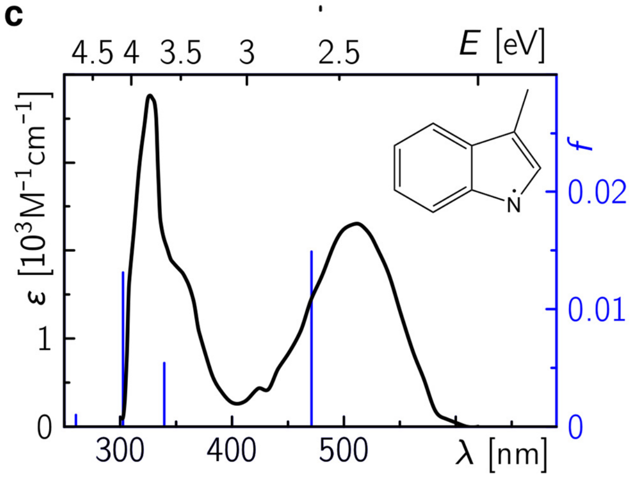

d

state/ transition[a]	ΔE [eV]	[nm]	f	μ_{ij} [D]	ϕ_y [°]
$\lvert D_0 \rangle$				2.8	10
$D_1 \leftarrow D_0$	1.23	1009	10^{-4}	0.2	117
$D_2 \leftarrow D_0$	2.63	471	0.015	1.2	100
$D_3 \leftarrow D_0$	3.65	339	0.005	0.6	110

[a]considering only π-π^* type

Fig. S8 Quantum chemical calculations of tryptophan neutral radical. a: Optimised geometry. **b**: Static dipole moment of ground state as well as most prominent transition dipole moments. Colour coding as in **d**. **c**: Experimental (black)[9] and theoretical (blue) absorption spectra. **d**: Details to dipole moments and transition dipole moments shown in **b** (Transition energy, ΔE; oscillator strength, f; static/transition dipole moments, μ_{ij}; angle to y-axis, ϕ_y).

a

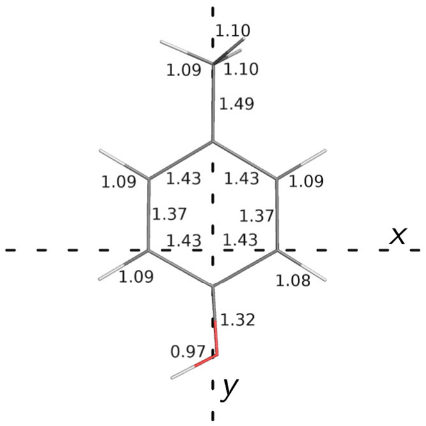

b

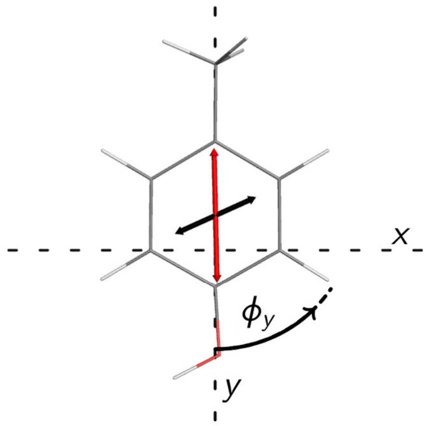

c

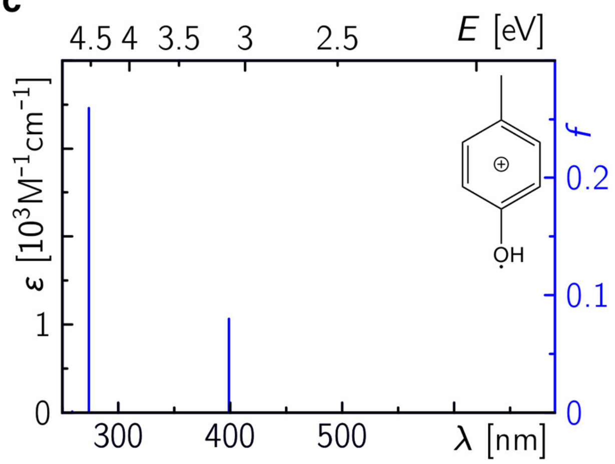

d

state/ transition[a]	ΔE [eV]	[nm]	f	μ_{ij} [D]	ϕ_y [°]
$\|D_0\rangle$				1.7	114
$D_1 \leftarrow D_0$	1.41	876	$5 \cdot 10^{-4}$	0.3	97
$D_2 \leftarrow D_0$	3.11	399	0.080	2.6	1
$D_3 \leftarrow D_0$	4.53	274	0.260	3.9	179

[a]considering only π-π^* type

Fig. S9 Quantum chemical calculations of tyrosine radical cation. a: Optimised geometry. **b**: Static dipole moment of ground state as well as most prominent transition dipole moments. Colour coding as in **d**. **c**: Theoretical (blue) absorption spectrum. **d**: Details to dipole moments and transition dipole moments shown in **b** (Transition energy, ΔE; oscillator strength, f; static/transition dipole moments, μ_{ij}; angle to y-axis, ϕ_y).

a **b**

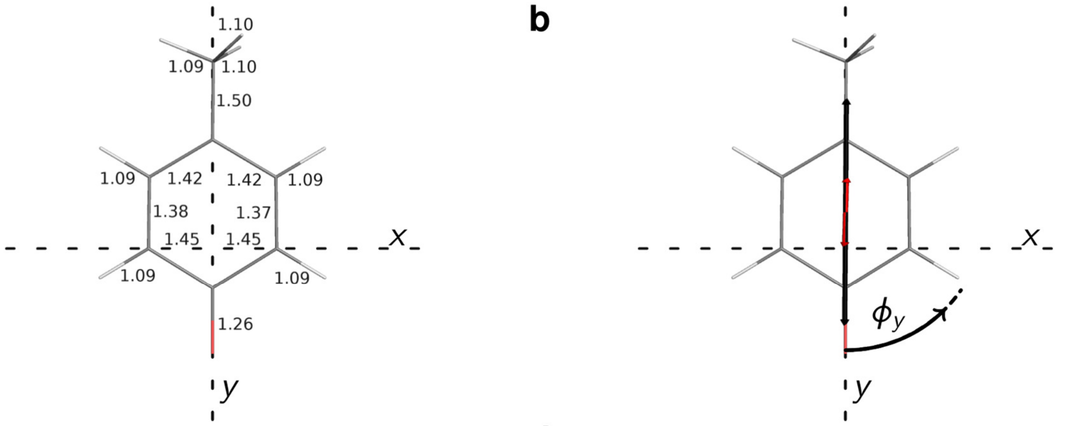

c

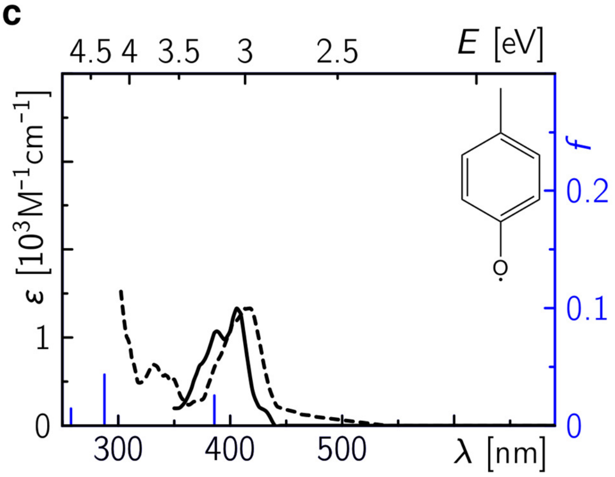

d

state/ transition[a]	ΔE [eV]	[nm]	f	μ_{ij} [D]	ϕ_y [°]
$\lvert D_0 \rangle$				4.5	179
$D_1 \leftarrow D_0$	2.39	517	0.003	0.5	93
$D_2 \leftarrow D_0$	3.21	386	0.025	1.4	178
$D_3 \leftarrow D_0$	4.31	288	0.043	1.6	83

[a]considering only π-π^* type

Fig. S10 Quantum chemical calculations of tyrosine neutral radical. a: Optimised geometry. **b**: Static dipole moment of ground state as well as most prominent transition dipole moments. Colour coding as in **d**. **c**: Experimental (black)[11] and theoretical (blue) absorption spectra. **d**: Details to dipole moments and transition dipole moments shown in **b** (Transition energy, ΔE; oscillator strength, f; static/transition dipole moments, μ_{ij}; angle to y-axis, ϕ_y).

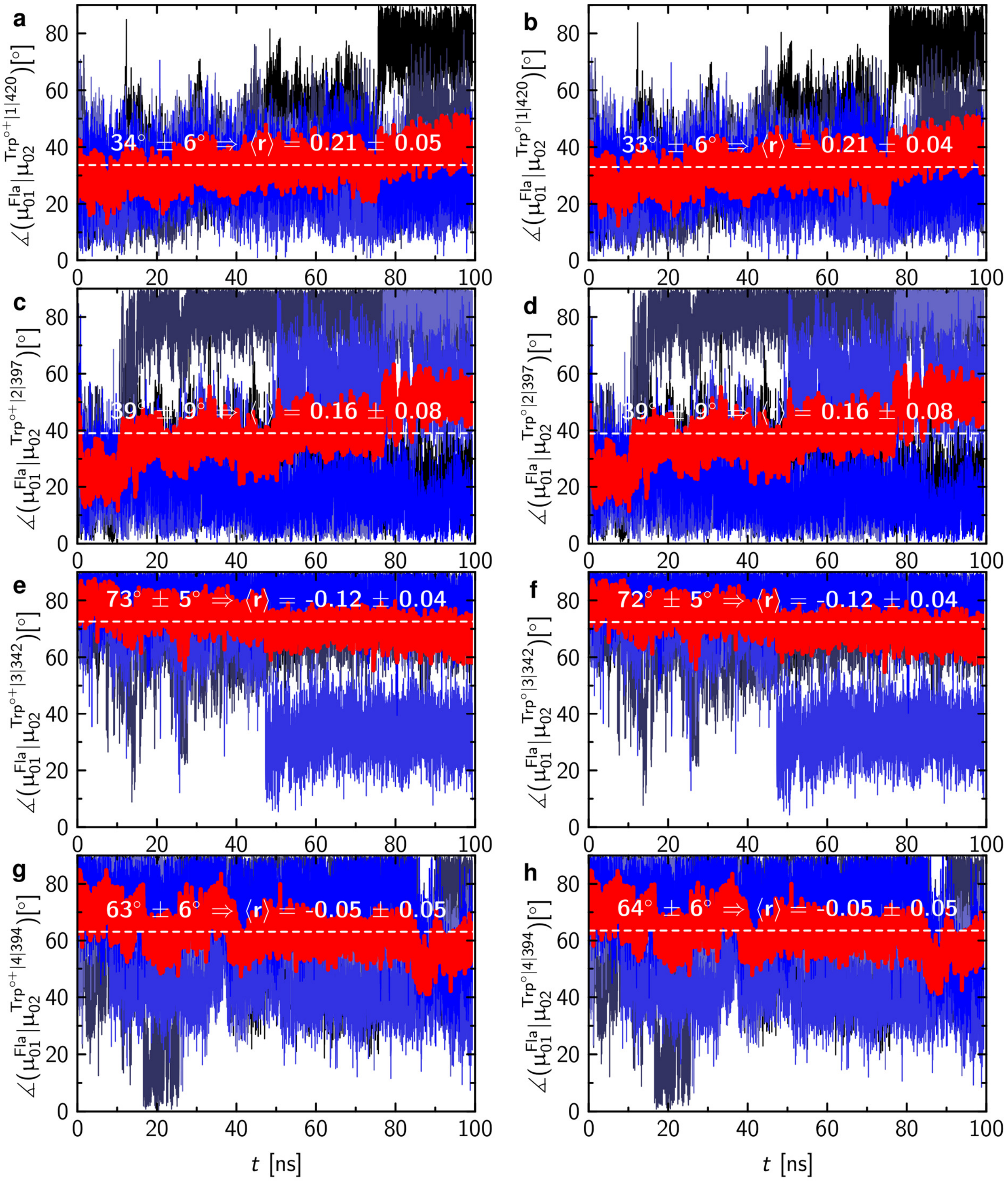

Fig. S11 Angles between the transition moment for flavin excitation, μ_{01}, to the transition moments of the most prominent transition dipole moments of all four tryptophans of the eT pathway in either $^{2}Trp^{\circ +}$ or $^{2}Trp^{\circ}$ form along five independent molecular dynamic simulations on DmCRY in its 'dark' ground state. a: ^{1}[FAD°-| $Trp_{420}^{(1)\circ +}$], **b:** ^{1}[FAD°-| $Trp_{420}^{(1)\circ}$], **c:** ^{1}[FAD°-| $Trp_{397}^{(2)\circ +}$], **d:** ^{1}[FAD°-| $Trp_{397}^{(2)\circ}$], **e:** ^{1}[FAD°-| $Trp_{342}^{(3)\circ +}$], **f:** ^{1}[FAD°-| $Trp_{342}^{(3)\circ}$], **g:** ^{1}[FAD°-| $Trp_{394}^{(4)\circ +}$], and **h:** ^{1}[FAD°-| $Trp_{394}^{(4)\circ}$]. Individual runs are shown in blueish tones and the average in red. The value shown gives the total average over time and all five runs.

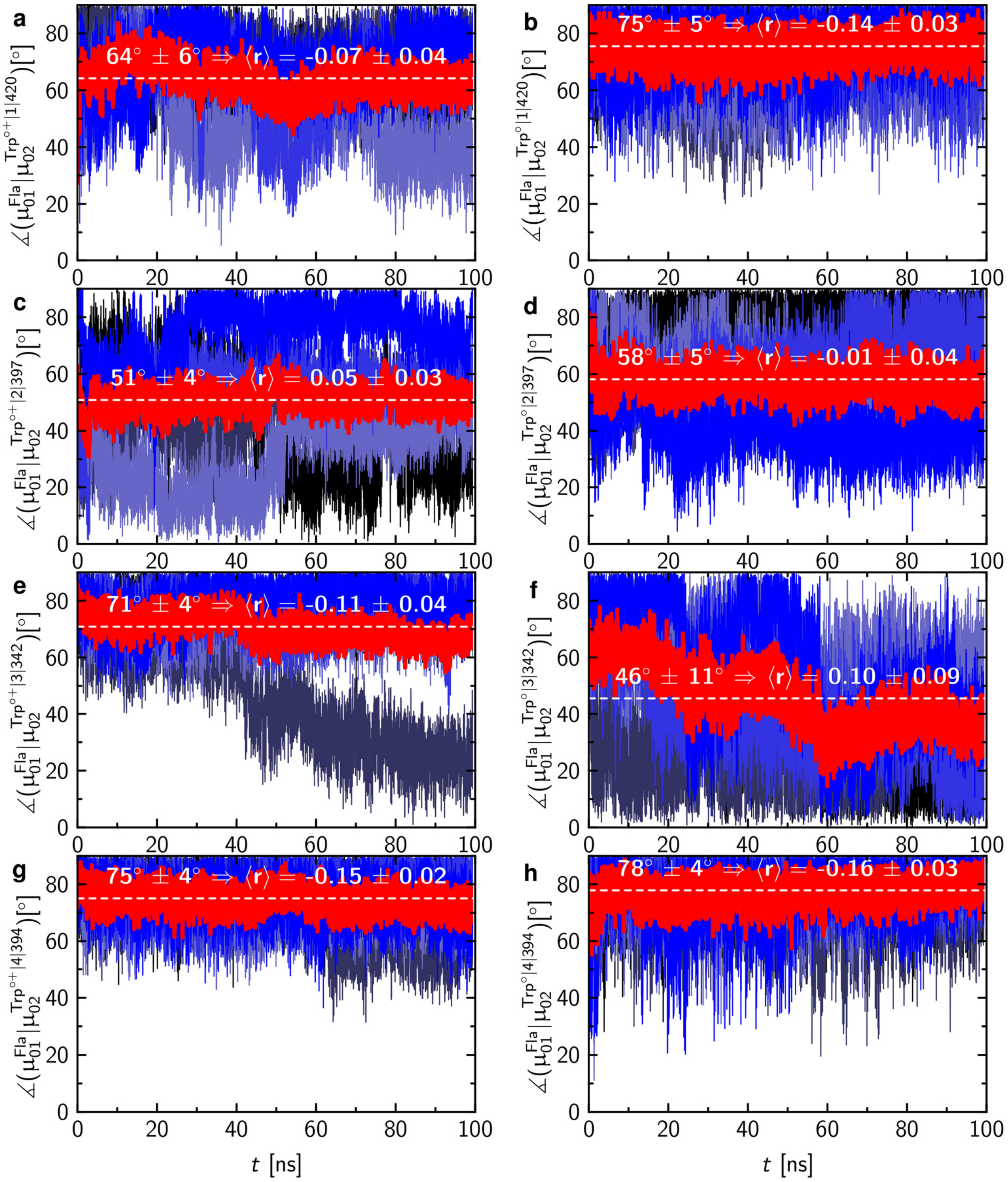

Fig. S12 Angles between the transition moment for flavin excitation, μ_{01}, to the transition moments of the most prominent transition dipole moments of all four tryptophans of the eT pathway in either ^{2}Trp°+ or ^{2}Trp° form along five independent molecular dynamic simulations on DmCRY in its signalling states. a: ^{1}[FAD°-| $\mathrm{Trp}_{420}^{(1)\,\circ+}$], **b:** ^{1}[FAD°-| $\mathrm{Trp}_{420}^{(1)\,\circ}$], **c:** ^{1}[FAD°-| $\mathrm{Trp}_{397}^{(2)\,\circ+}$], **d:** ^{1}[FAD°-| $\mathrm{Trp}_{397}^{(2)\,\circ}$], **e:** ^{1}[FAD°-| $\mathrm{Trp}_{342}^{(3)\,\circ+}$], **f:** ^{1}[FAD°-| $\mathrm{Trp}_{342}^{(3)\,\circ}$], **g:** ^{1}[FAD°-| $\mathrm{Trp}_{394}^{(4)\,\circ+}$], and **h:** ^{1}[FAD°-| $\mathrm{Trp}_{394}^{(4)\,\circ}$]. Individual runs are shown in blueish tones and the average in red. The value shown gives the total average over time and all five runs.

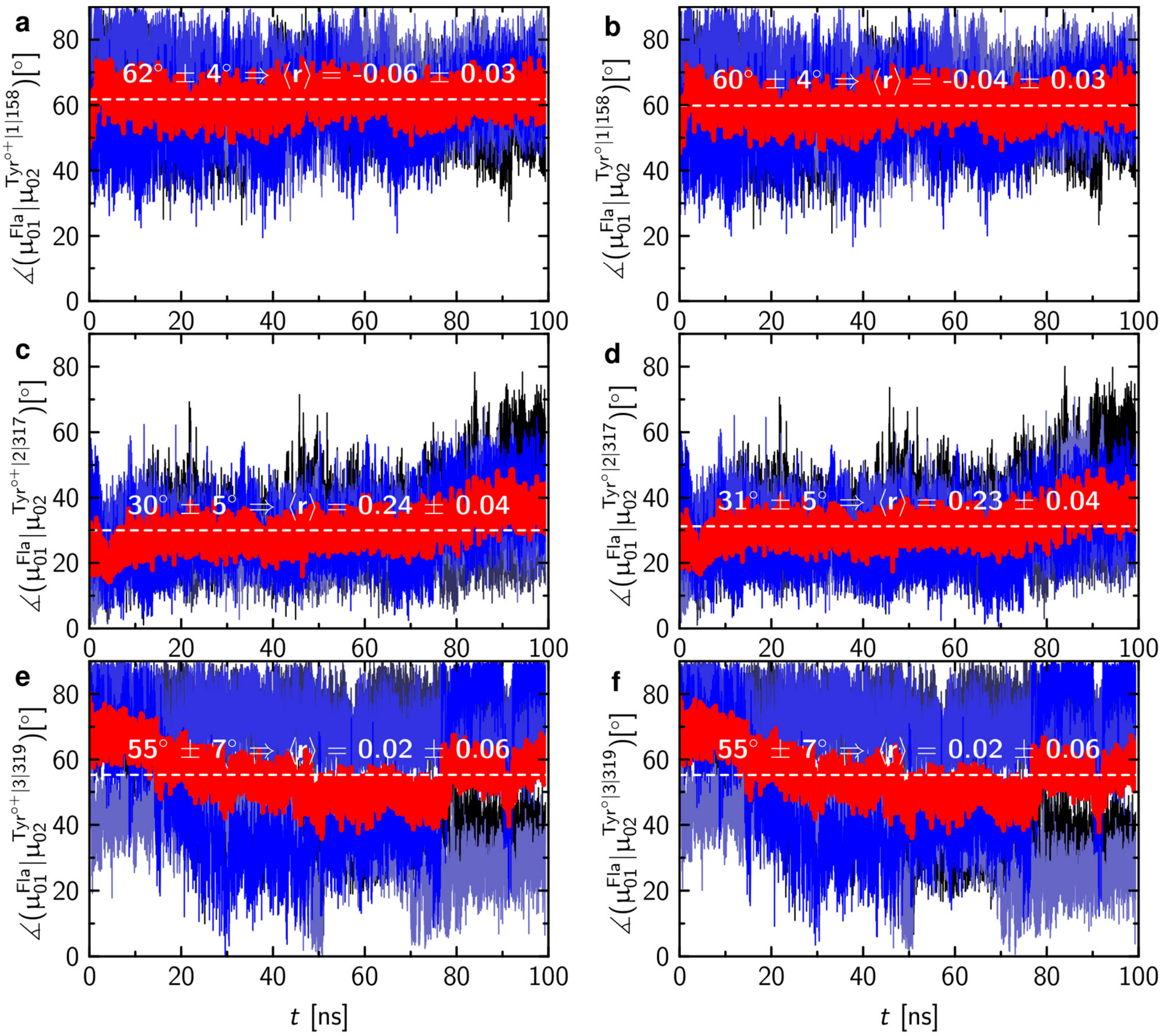

Fig. S13 Angles between the transition moment for flavin excitation, μ_{01}, to the transition moments of the most prominent transition dipole moments of all three tyrosines of the alternative eT pathway in either $^{2}Tyr^{\circ+}$ or $^{2}Tyr^{\circ}$ form along five independent molecular dynamic simulations on DmCRY in its 'dark' ground state. a: ^{1}[FAD°⁻| $Tyr_{158}^{(1)\,\circ+}$], **b:** ^{1}[FAD°⁻| $Tyr_{158}^{(1)\,\circ}$], **c:** ^{1}[FAD°⁻| $Tyr_{317}^{(2)\,\circ+}$], **d:** ^{1}[FAD°⁻| $Tyr_{317}^{(2)\,\circ}$], **e:** ^{1}[FAD°⁻| $Tyr_{319}^{(3)\,\circ+}$], and **f:** ^{1}[FAD°⁻| $Tyr_{319}^{(3)\,\circ}$]. Individual runs are shown in blueish tones and the average in red. The value shown gives the total average over time and all five runs.

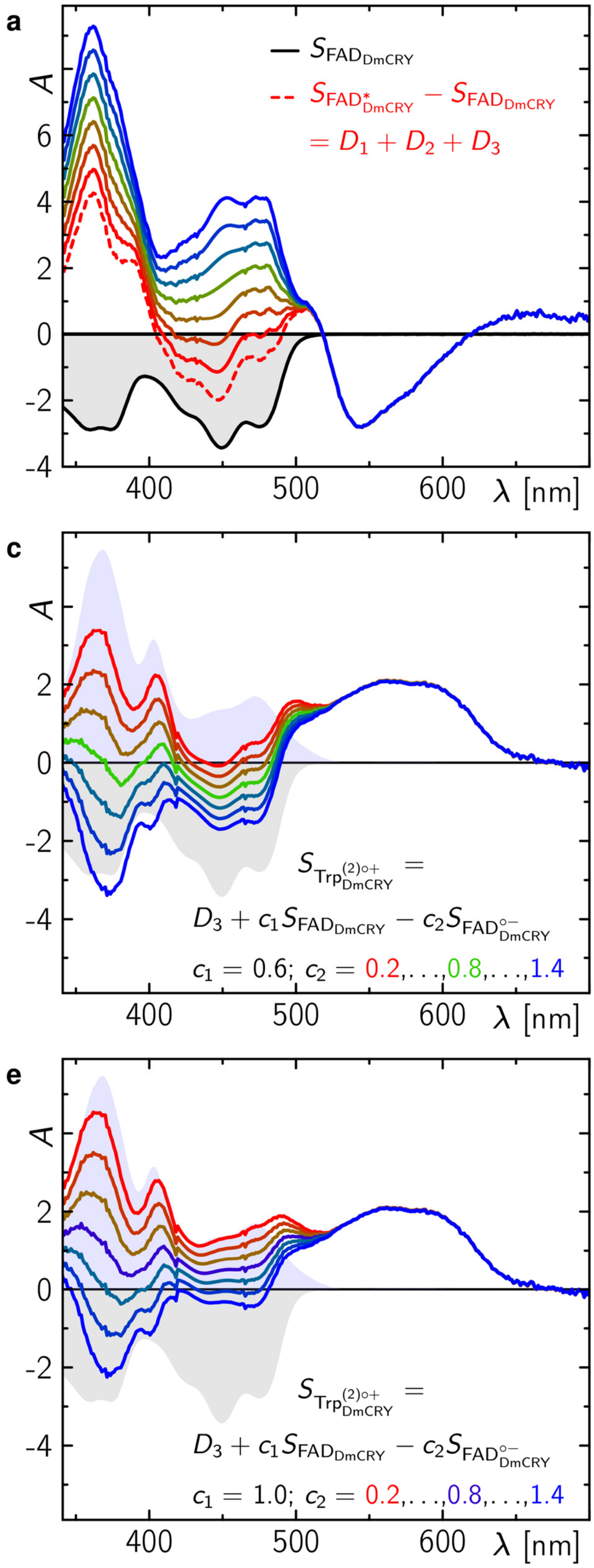

Fig. S14 Determination of the $Trp^{(2)}{}_{DmCRY}{}^{\circ+}$ spectrum. a: Sum of Decay associated difference spectra from global fit on data in Fig. 2 ($D_1 + D_2 + D_3$, red dashed) and pure spectra for FAD_{DmCRY} ($S_{FAD_{DmCRY}}$, black). The FAD_{DmCRY} is multiplied by −1. *In silico* titration of ground state spectrum is shown. **b-f**: Linear combinations of D_3, $S_{FAD_{DmCRY}}$, and $S_{FAD_{DmCRY^{\circ-}}}$ for various weights as indicated.

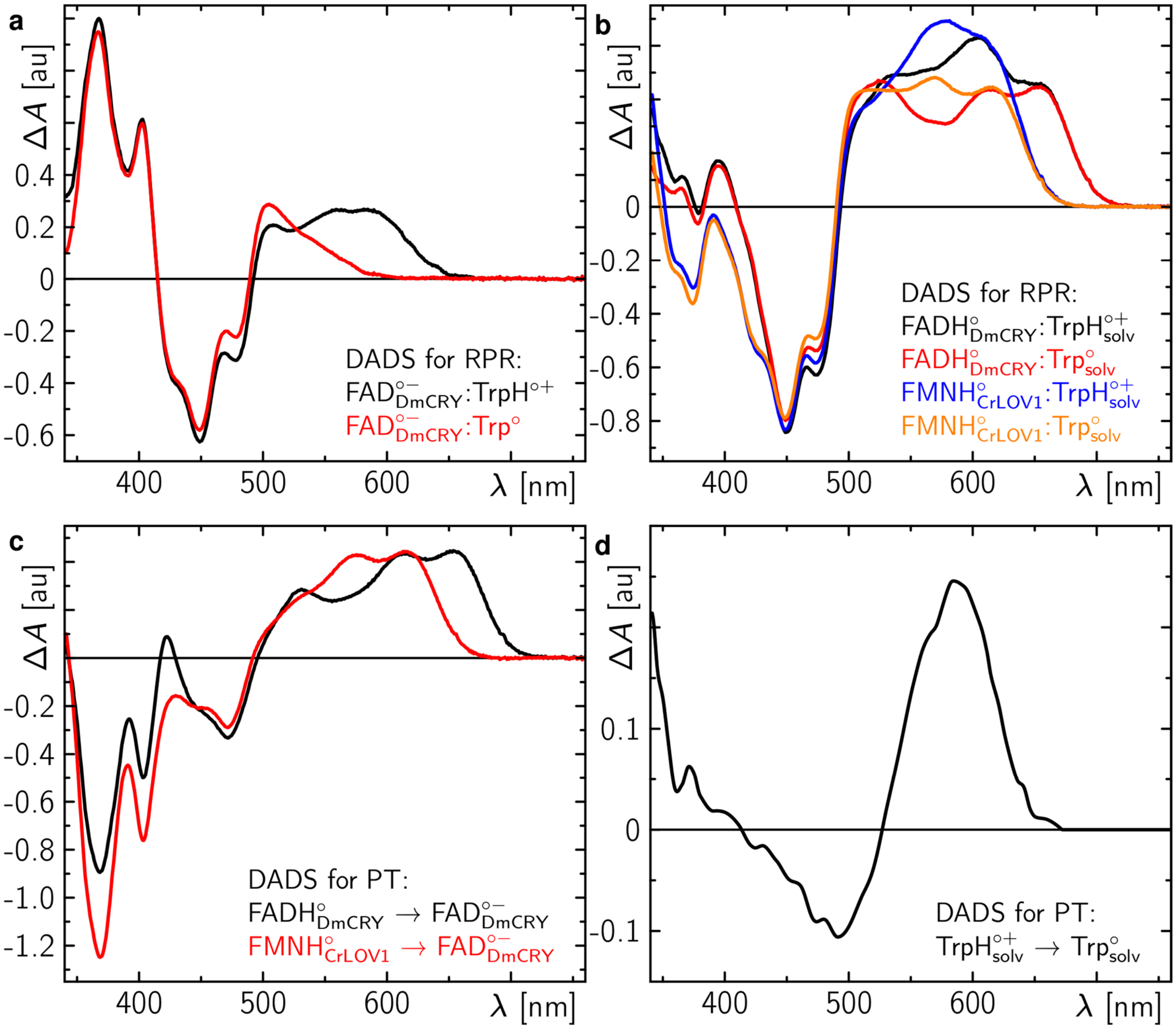

Fig. S15 Expected decay associated difference spectra (DADS) for either radical pair recombination (RPR) or proton transfer (PT) between all possible FAD and Trp radicals. a-b: RPR of pairs as indicated in each panel. **c-d**: PT of pairs as indicated in each panel.

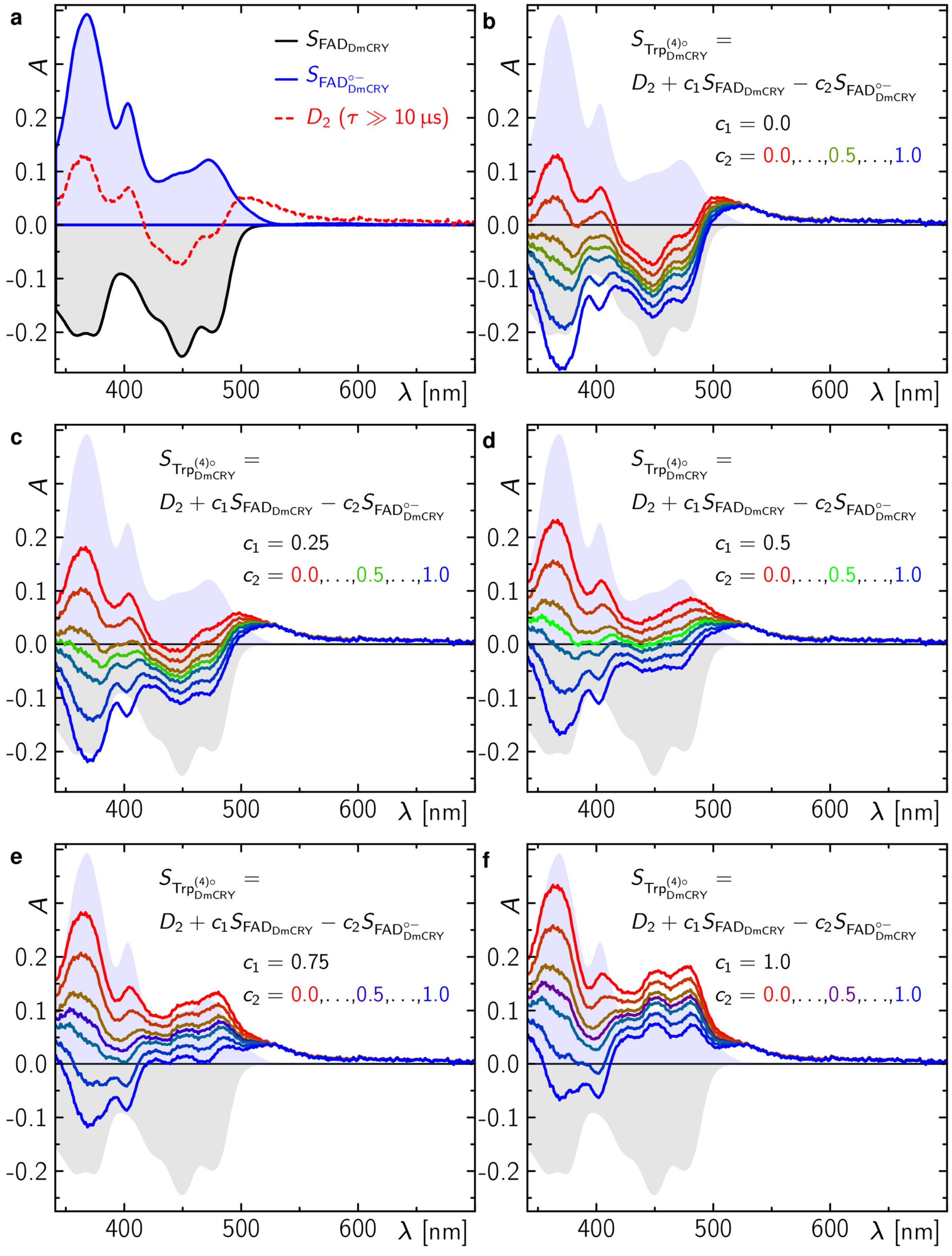

Fig. S16 Determination of the TrpDmCRY° spectrum. a: Decay associated difference spectrum from global fit on data in Fig. 3 (D_2, red dashed) and pure spectra for FADDmCRY ($S_{FAD_{DmCRY}}$, black) and FADDmCRY°⁻ ($S_{FAD_{DmCRY^{\circ-}}}$, blue). The FADDmCRY is multiplied by −1. **b-f**: Linear combinations of D_2, $S_{FAD_{DmCRY}}$, and $S_{FAD_{DmCRY^{\circ-}}}$ for various weights as indicated.

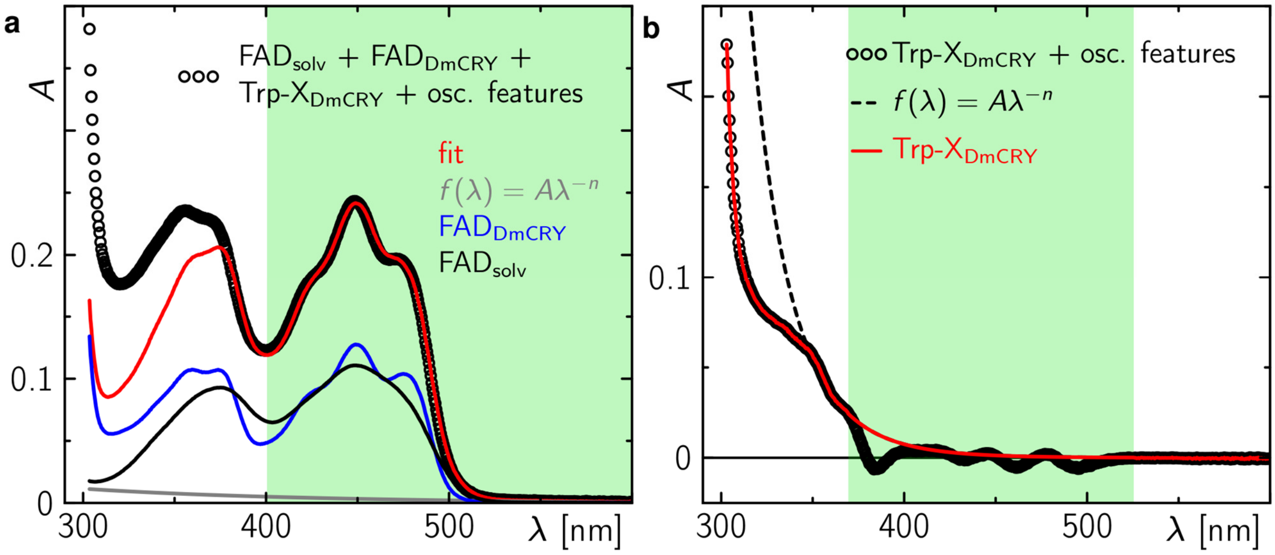

Fig. S17 Extraction of Trp-X$_{DmCRY}$ spectrum from stationary illumination data. a: Fit of FAD$_{DmCRY}$ dark state spectrum (blue line) from freshly purified sample in the dark, FAD$_{solv}$ spectrum (black line) recorded in this work, and a scatter function (grey line) to the last spectrum of the sequence shown in Figure 4 (black open circles). **b**: Correction for small oscillatory features due to loss in fine structure signatures of the FAD$_{solv}$ spectrum. The green background indicates the data range included in the fit.

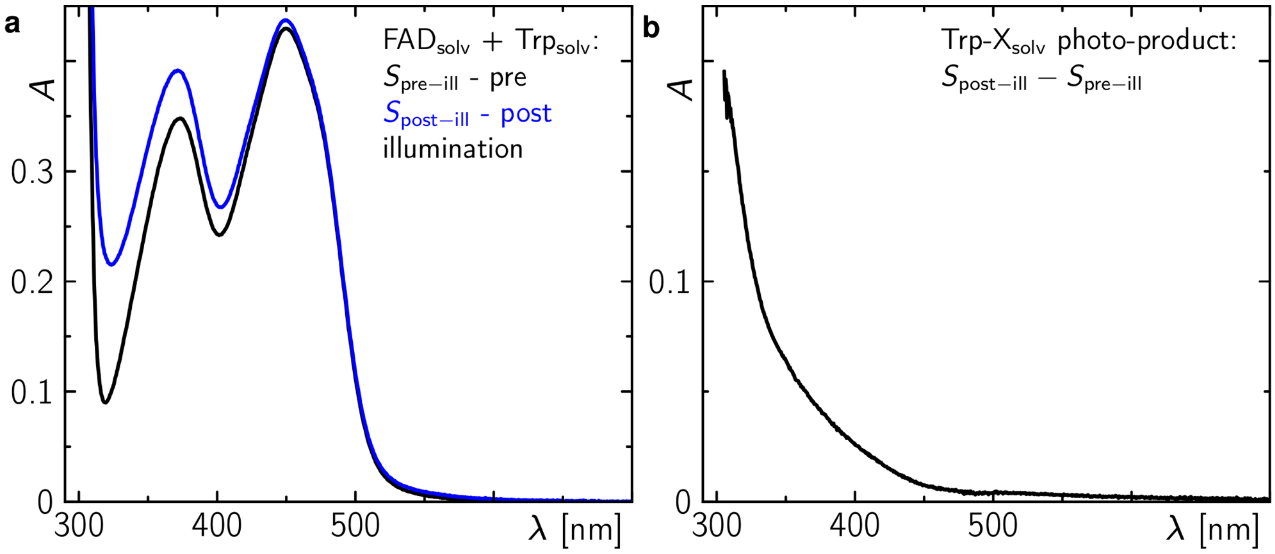

Fig. S18 Irreversible tryptophan decomposition by flavin photocatalysis. a: Stationary absorption spectra of a mixture of FAD (200 μM) and Trp (41 mM) in buffer (50 mM HEPES, 150 mM NaCl, 10 Vol% glycerol, pH = 8.0) prior (black) and post (blue) illumination with blue light (450 nm). **b**: Difference absorption spectrum of spectra in **a** showing the irreversible Trp photo-product.

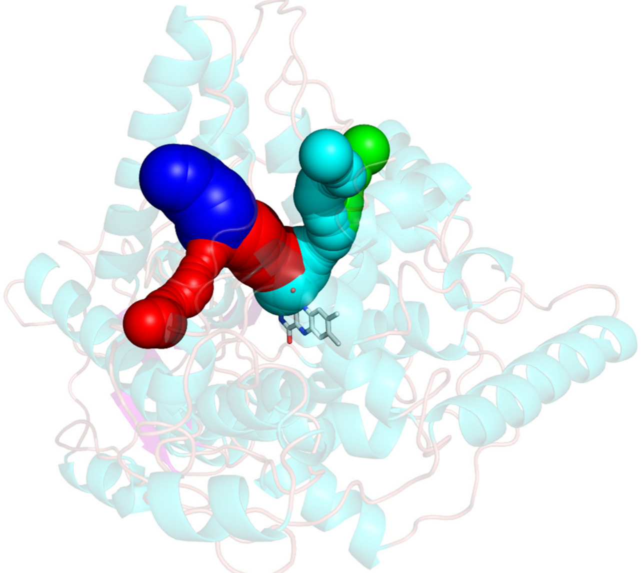

Fig. S19 Molecular tunnels from the surface to the DmCRY FAD binding pocket. DmCRY (pdb code: 4GU5)[5] secondary structure (transparent cartoon) with highlighted FAD in stick representation and four potential tunnels allowing entry of small molecules into the FAD binding pocket. The tunnels were calculated by the Caver 3.0 program.[12]

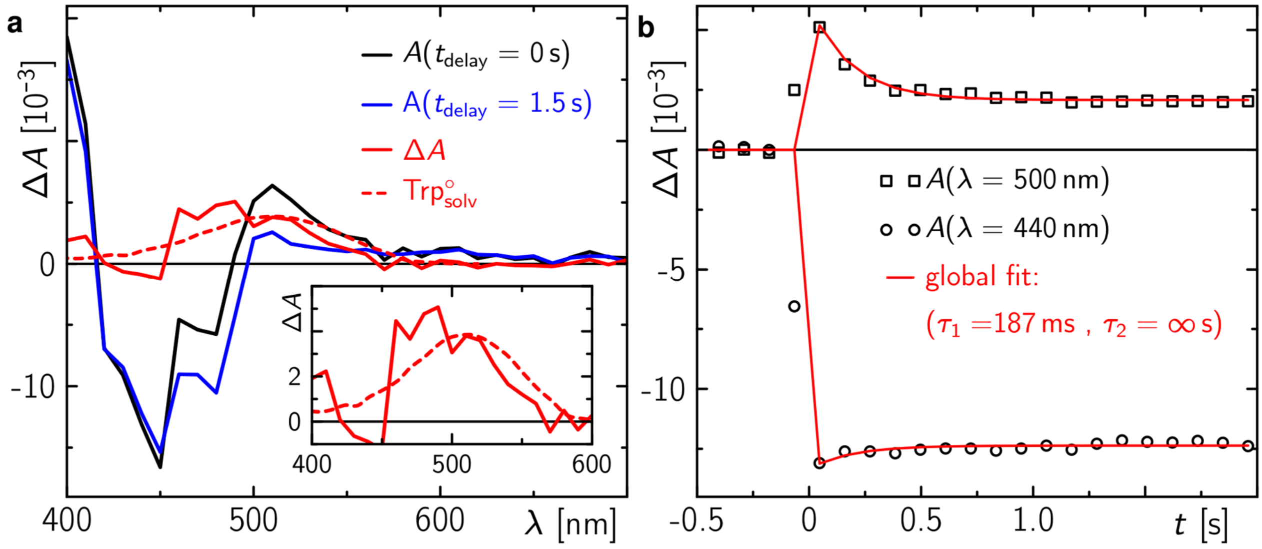

Fig. S20 Decoupled tryptophanyl radical decomposition. a: Selected time-resolved absorption data in the ms to min time range at delay times as indicated (black and blue lines). The difference spectrum of these (red line) resembles the known reference spectrum[9] of Trp°_{solv} (red dashed line). **b**: Selected time traces at wavelength as indicated (open circles and squares). The red lines show the bi-exponential global fit to the data with lifetimes as indicated.

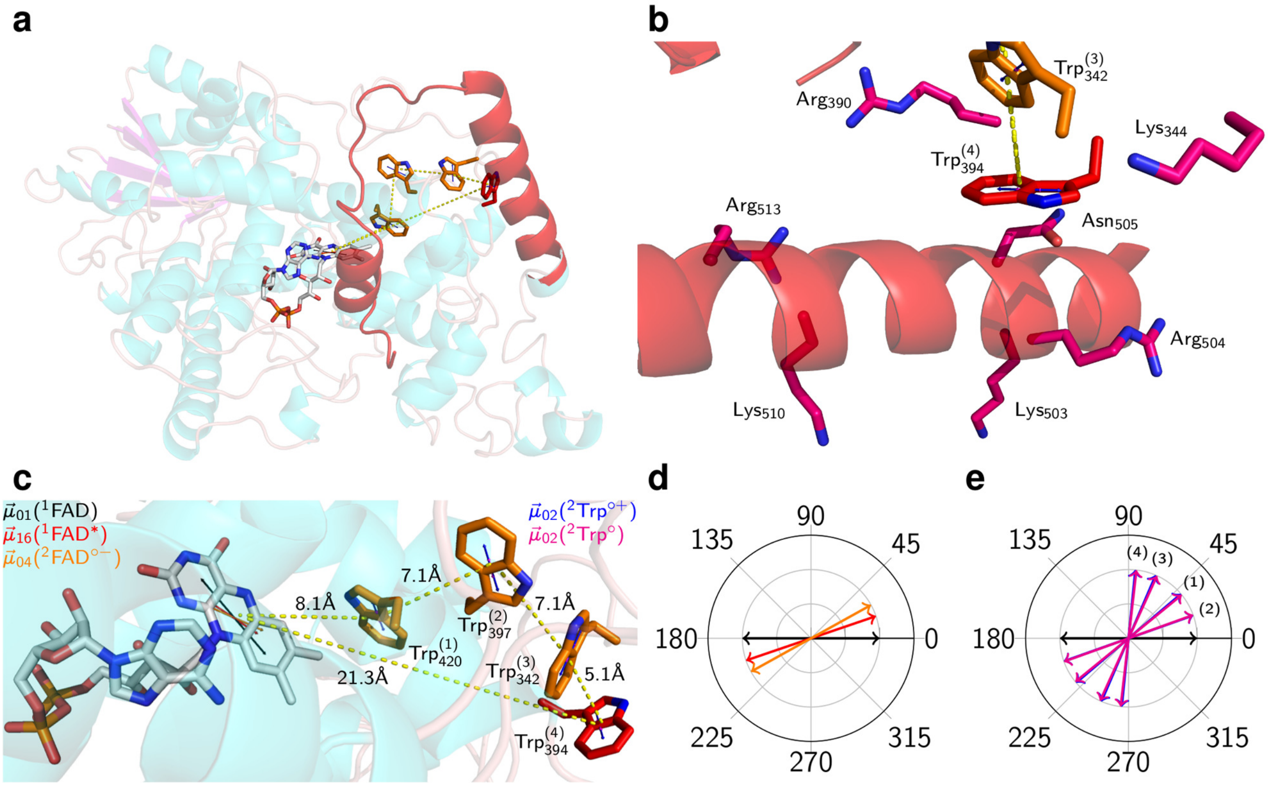

Fig. S21 Geometry of the reaction centre in DmCRY. a: global overview of DmCRY (pdb code: 4GU5)[5] secondary structure (transparent cartoon) with highlighted C-terminus connected to helix $\alpha 22$ (red cartoon), FAD (grey carbons), $Trp_{420}^{(1)}$ (orange carbons), $Trp_{397}^{(2)}$ (orange carbons), $Trp_{342}^{(3)}$ (orange carbons), $Trp_{394}^{(4)}$ (red carbons). **b**: Zoom into the surface exposed $Trp_{394}^{(4)}$ (red carbons) and its interaction to helix $\alpha 22$ that directly connects to the C-terminus. Positively charged amino acids are shown in stick representation. **c**: Zoom into the FAD binding pocket and the adjacent eT pathway along the tryptophan tetrad. **d & e**: Angles between the transition moment for flavin excitation, μ_{01}, to the transition moments of all prominent absorption bands as indicated in **c**.

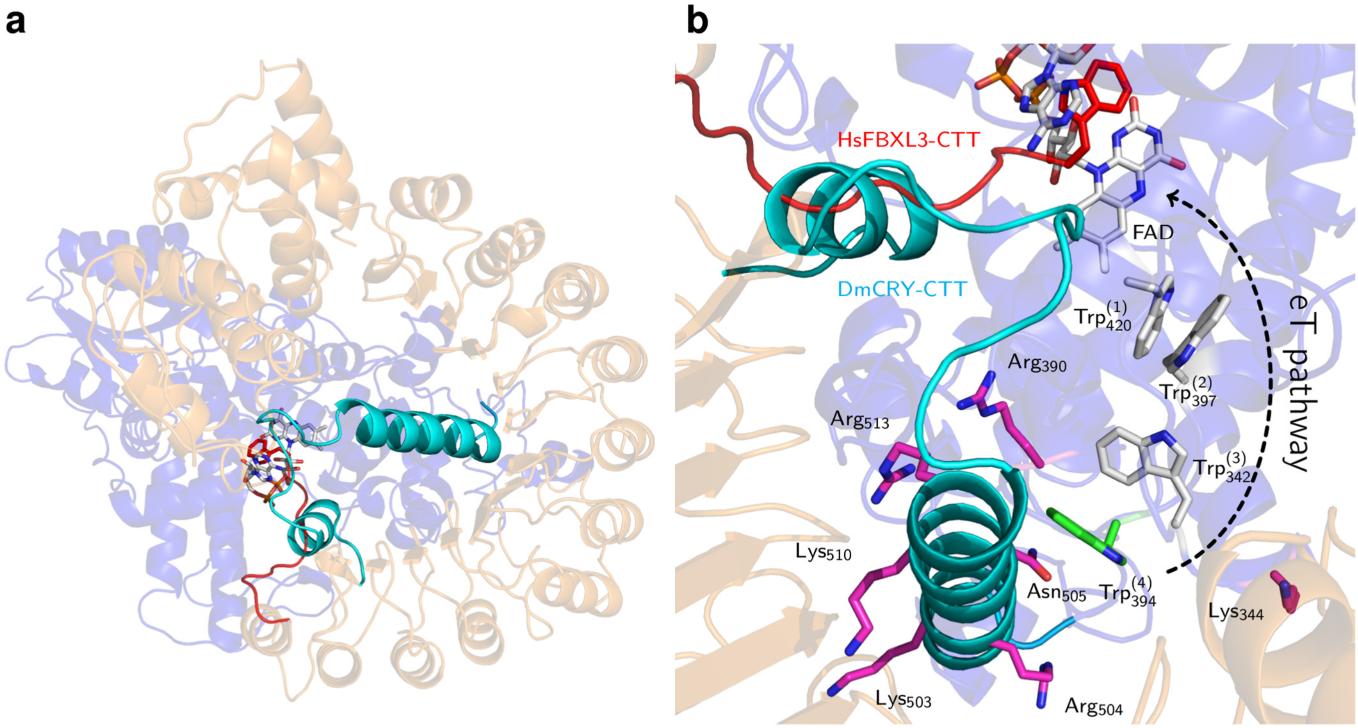

Fig. S22 Hypothetical DmCRY/FBXL3-complex. **a**: Overview of the DmCRY/FBXL3-complex (PDB codes: 4GU5 (blue, DmCRY)[5] and 4I6J (orange, MmCRY2/FBXL3)[13]). DmCRY is aligned to MmCRY2 (not shown). The C-terminal tails (CTT) of DmCRY and FBXL3 are highlighted in cyan and red, respectively. **b**: Zoom into the FAD binding pocket and the adjacent eT pathway along the tryptophan tetrad and interaction between helix $\alpha22$ and the surface exposed $Trp^{(4)}_{394}$. FAD, tryptophan tetrad, and positively charged amino acids are shown in stick representation.

References

[1] A. Jablonski, *Zeitschrift für Physik A Hadrons and Nuclei* **1936**, *103*, 526–535.
[2] J. R. Lakowicz, *Principles Fluorescence Spectroscopy*, **2006**.
[3] N.-S. Cheng, *Industrial & Engineering Chemistry Research* **2008**, *47*, 3285–3288.
[4] A. Volk, C. J. Kähler, *Experiments in Fluids* **2018**, *59*, 75.
[5] C. Levy, B. D. Zoltowski, A. R. Jones, A. T. Vaidya, D. Top, J. Widom, M. W. Young, N. S. Scrutton, B. R. Crane, D. Leys, *Nature* **2013**, *495*, E3–E4.
[6] R. J. Kutta, N. Archipowa, L. O. Johannissen, A. R. Jones, N. S. Scrutton, *Scientific Reports* **2017**, *7*, 44906.
[7] N. Öztürk, C. P. Selby, S.-H. Song, R. Ye, C. Tan, Y.-T. Kao, D. Zhong, A. Sancar, *Biochemistry* **2009**, *48*, 8585–8593.
[8] T. Climent, R. González-Luque, M. Merchán, L. Serrano-Andrés, *The Journal of Physical Chemistry A* **2006**, *110*, 13584–13590.
[9] S. Solar, N. Getoff, P. S. Surdhar, D. A. Armstrong, A. Singh, *The Journal of Physical Chemistry* **1991**, *95*, 3639–3643.
[10] R. Martin, F. Lacombat, A. Espagne, N. Dozova, P. Plaza, J. Yamamoto, P. Muller, K. Brettel, A. de la Lande, *Physical Chemistry Chemical Physics* **2017**, *19*, 24493–24504.
[11] S. Oldemeyer, S. Franz, S. Wenzel, L.-O. Essen, M. Mittag, T. Kottke, *Journal of Biological Chemistry* **2016**, *291*, 14062–14071.
[12] E. Chovancova, A. Pavelka, P. Benes, O. Strnad, J. Brezovsky, B. Kozlikova, A. Gora, V. Sustr, M. Klvana, P. Medek, et al., *PLOS Computational Biology* **2012**, *8*, 1–12.
[13] W. Xing, L. Busino, T. R. Hinds, S. T. Marionni, N. H. Saifee, M. F. Bush, M. Pagano, N. Zheng, *Nature* **2013**, *496*, 64–68.
